# Supplementary material for: Usp25-Erlin1/2 activity limits cholesterol flux to restrict virus infection
Source: Dev Cell. 2023 Nov 20;58(22):2495–2509.e6. doi: 10.1016/j.devcel.2023.08.013 (PMC10914638; doi:10.1016/j.devcel.2023.08.013)
Supplement: Document S1. Figures S1–S7 [file mmc1.pdf]

Developmental Cell, Volume *58*

Supplemental information

Usp25-Erlin1/2 activity limits cholesterol flux to restrict virus infection

Qi Wen Teo, Ho Him Wong, Tiaan Heunis, Viktoriya Stancheva, Asmaa Hachim, Huibin Lv, Lewis Siu, Julian Ho, Yun Lan, Chris Ka Pun Mok, Rachel Ulferts, and Sumana Sanyal

Supplementary Text

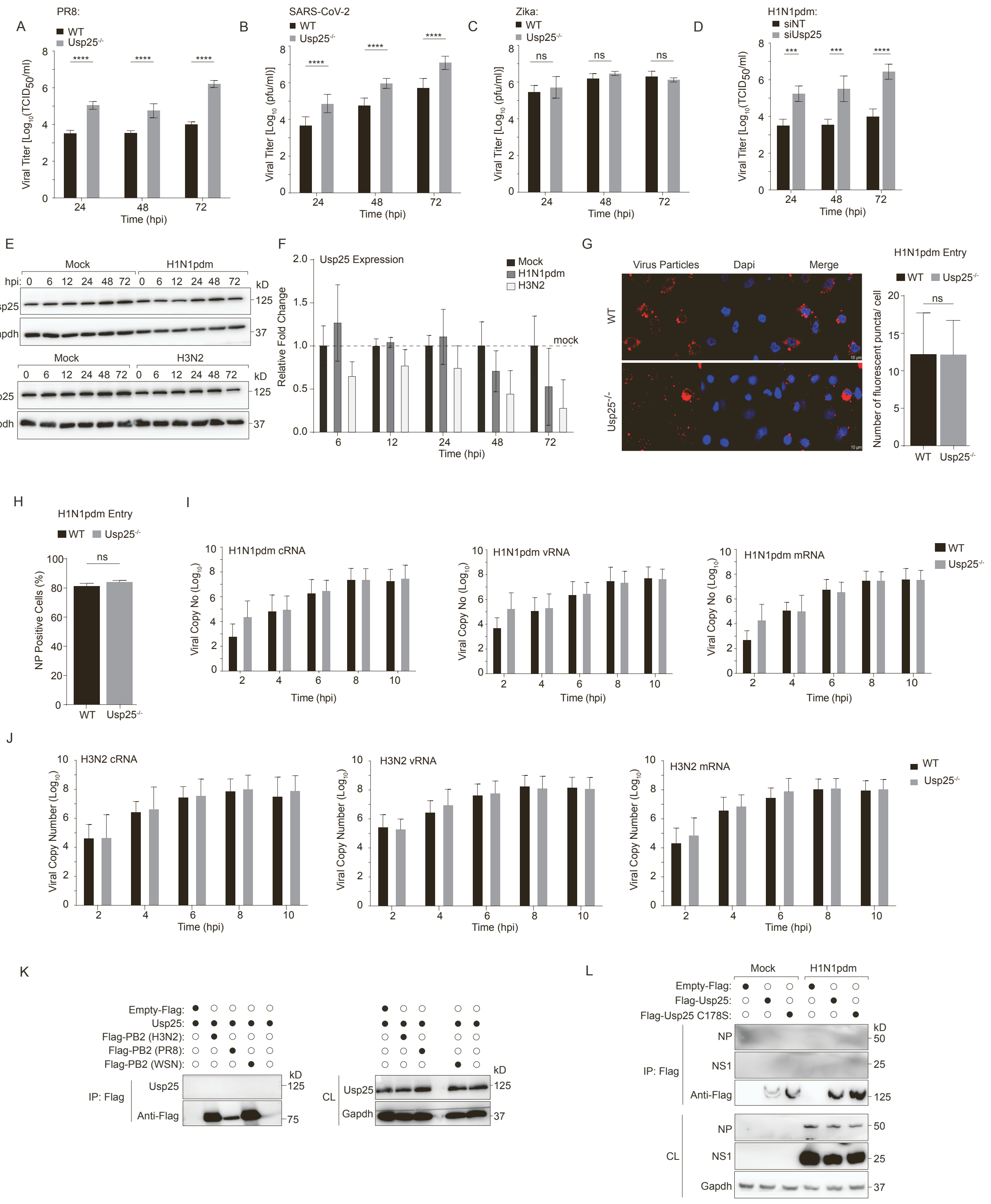

Figure S1. Usp25-deficiency does not affect viral entry and replication, related to Figure 1

(A-C) Viral titers measured from WT and Usp25$^{-/-}$ A549 cells infected with IAV PR8 (A), SARS-CoV-2 (B) and Zika (C) at the indicated timepoints (MOI = 0.1). Viral titers were determined by

plaque assay on MDCK and Vero cells respectively. Data are shown as means ± SD of three independent experiments. [****$p<0.0001$ analyzed by one-way ANOVA].

(D) Primary human bronchial epithelial cells from ATCC were treated with control siRNA (siNT) or Usp25 (siUSP25) and challenged with H1N1pdm (MOI 0.01). At indicated time points, viral titres were measured using TCID50/ml. Data are shown as mean ± SD of three independent experiments. [****$p<0.0001$ by one-way ANOVA].

(E) Western blot analysis of Usp25 protein expression upon IAV infection in WT A549 cells at the indicated timepoints; MOI = 0.01 used for multiple infection cycles; and MOI = 5 for single infection cycle. Gapdh was used as loading control. **(F)** Protein expression was quantified by densitometry and normalized to Gapdh levels. Data are shown as means of n=3 ± standard deviations (SD). Data analyzed by two-way ANOVA.

(G; *left panel***)** Immunofluorescence images of A549 cells infected with R18-labelled H1N1pdm virus (MOI=10). Viral particles are depicted in red. (*Right panel*) Viral entry was quantified by calculating the number of internalized viral particle per cell. Data are shown as means of n=50 ± standard deviation (SD). Data analyzed by Student's unpaired T test; ns: not significant.

(H) WT and Usp25$^{-/-}$ A549 cells were infected with H1N1pdm virus and the infected positive cells for NP were quantified by flow cytometry (MOI= 5). Data are shown as means of n=3 ± standard deviations (SD). Data analyzed by Student's unpaired T test; ns: not significant.

(I, J) cRNA, vRNA, mRNA levels of the Influenza M1 gene after H1N1pdm infection (I) or H3N2 infection (J) in A549 cells (MOI=5). Samples were collected at the indicated timepoints and were analyzed by real-time RT-qPCR. The specificities of the amplified products were confirmed by the melting curve analysis. Data are shown as means of n=3 ± standard deviation (SD). Data analyzed by two-way ANOVA.

(K) HEK-293T cell extracts, transiently co-transfected with Usp25 together with the indicated plasmids encoding the Flag-PB2 from H3N2, PR8, or WSN IAV strains were immunoprecipitated on anti-Flag M2 affinity beads. The immunoprecipitates (IP) were analyzed by immunoblotting (IB) with anti-Usp25 and anti-Flag antibodies. Transfected USP25 was detected by immunoblotting the cell lysates (CL) with anti-Usp25. Gapdh was used as loading control.

(L) H1N1pdm infected HEK-293T cell extracts, transiently transfected with Flag-Usp25 or Flag-USP25 C178S expression constructs were immunoprecipitated on anti-Flag M2 affinity beads. The immunoprecipitates (IP) were analyzed by immunoblotting (IB) with anti-NP and anti-NS1 antibodies. NP and NS1 expression were detected by immunoblotting the cell lysates (CL) with respective antibodies. Gapdh was used as loading control.

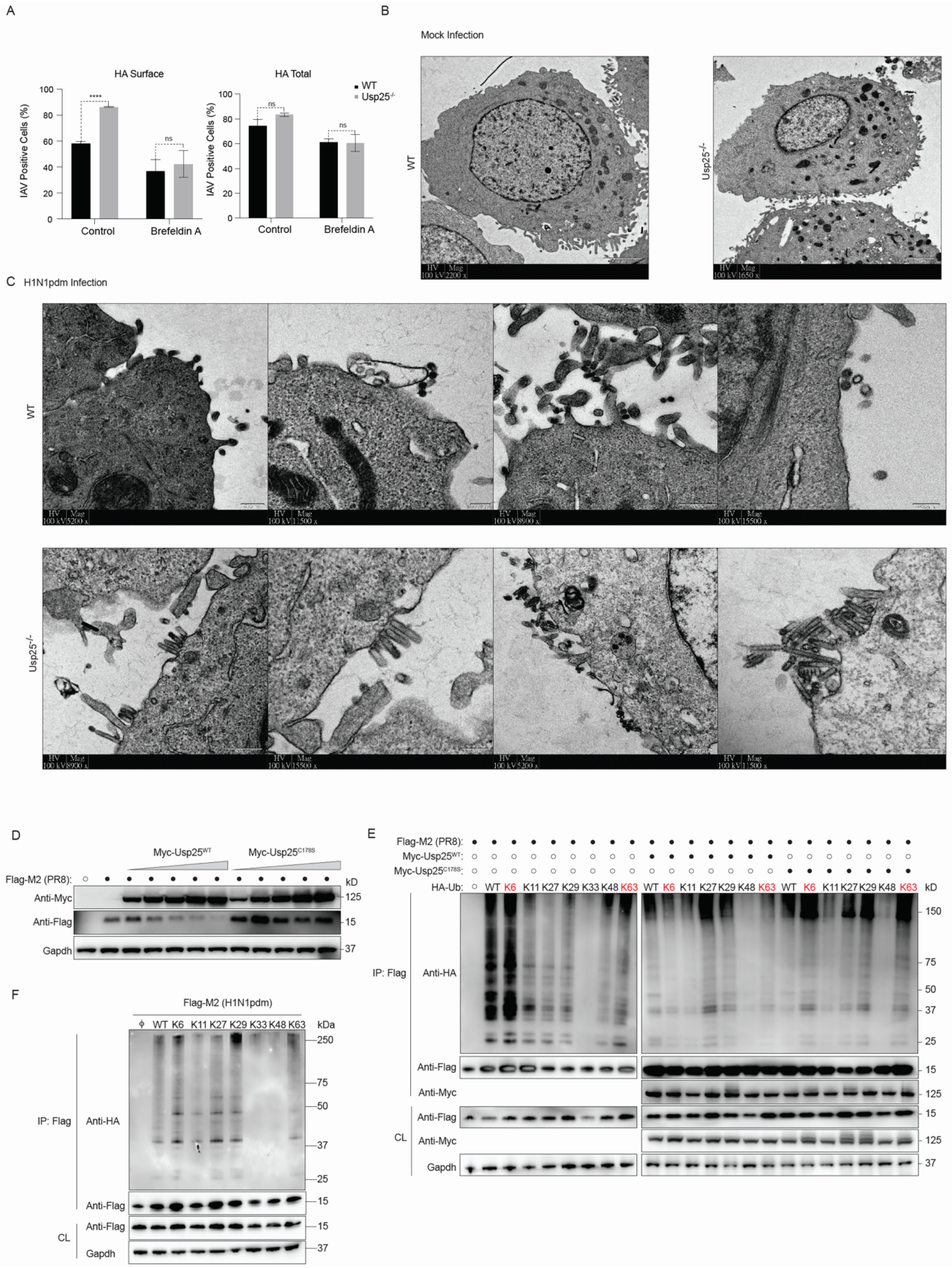

Figure S2. Usp25 alters M2-dependent budding of viral progenies, related to Figure 2

(A) IAV-infected WT and Usp25$^{-/-}$ A549 cells (MOI=5) were treated with Brefeldin A and total versus surface levels of viral HA was measure using flow cytometry. Data are shown as means of n=3 ± standard deviations (SD). Data analyzed by Student's unpaired T test was [**p<0.01, ***p<0.001].

(B, C) Negative stain EM of mock and H1N1pdm infected cells. (**D**) HEK293T cells co-transfected with Flag-M2 and Myc-Usp25^{WT} or Myc-Usp25^{C178S}. Expression of M2 and Usp25 were measured by immunoblotting. Anti-Myc was used to verified Usp25, and anti-Flag for M2. Gapdh was used as loading control**. (E)** HEK293T cells were co-transfected with Flag M2 and either myc-Usp25^{WT} or myc-Usp25^{C178S} along with HA-tagged ubiquitin (wild-type versus linkage specific constructs as indicated). M2 was immunoprecipitated on anti-Flag M2 affinity beads antibodies followed by immunoblotting with anti-HA to determine ubiquitylation. Anti-Myc was used to verified Usp25, and anti-Flag for M2. Gapdh was used as loading control. **(F)** Same as (E) using M2 from H1N1pdm IAV-strain.

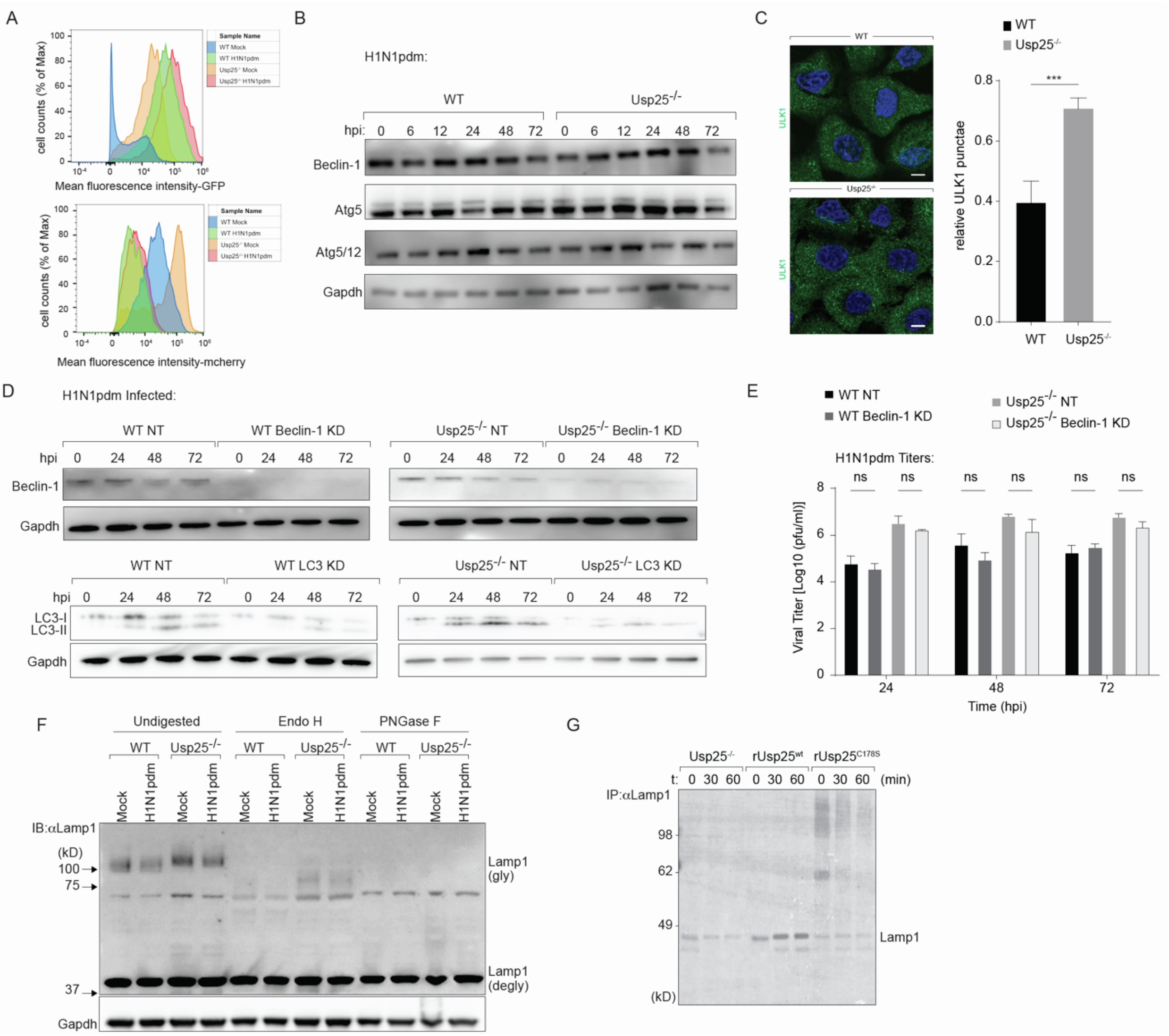

Figure S3. Usp25-deficient cells facilitate virus production via LC3 and Rab11 compartments, related to Figure 3

(A) Quantitation of mcherry and GFP populations of LC3 in mock and infected A549 cells. (**B**) WT A549 cells and Usp25$^{-/-}$ cells were infected with H1N1pdm (MOI=0.01). Total cell lysates were collected at the indicated time points, and protein expression of the autophagy markers were determined by Western blots. Gapdh levels measured as a loading control.

(**C**) ULK1 was visualized by confocal imaging in WT and Usp25$^{-/-}$ A549 cells. Quantitation of ULK1 punctae was calculated using “Analyze Particles” macro in Image J software. The quantitation was performed from three independent experiments with a sample size of 100 cells for each condition used to calculate statistical significance.

(**D**) Validation of Beclin1 and LC3 depletion was performed by immunoblotting in H1N1pdm-infected WT and Usp25$^{-/-}$ A549 cells at indicated timepoints. (MOI=0.01). Gapdh levels measured as a loading control.

(**E**) Viral titers from WT and Usp25$^{-/-}$ A549 cells depleted in Beclin1 at indicated timepoints were measured by plaque assay. (MOI=0.01). Data are shown as means of n=3 ± standard deviations (SD). Two-way ANOVA was used to analyze data [ns: not significant].

(**F, G**) Western blot analysis of Lamp1 glycosylation in WT and Usp25$^{-/-}$ A549 cells. Cells were infected with H1N1pdm (MOI= 0.01). Cell lysates were harvested and digested with either EndoH to cleave high-mannose and hybrid-mannose from *N*-linked glycoproteins or PNGase to remove all *N*-glycans for an hour. Gapdh was used as a loading control. (F) Usp25$^{-/-}$, Usp25^{WT} and Usp25^{C178S} were pulsed with [^{35}S]cysteine/methionine and chased for indicated time intervals. Lamp1 was immunoprecipitated on anti-Lamp1 antibodies, resolved by gel electrophoresis and detected by autoradiography.

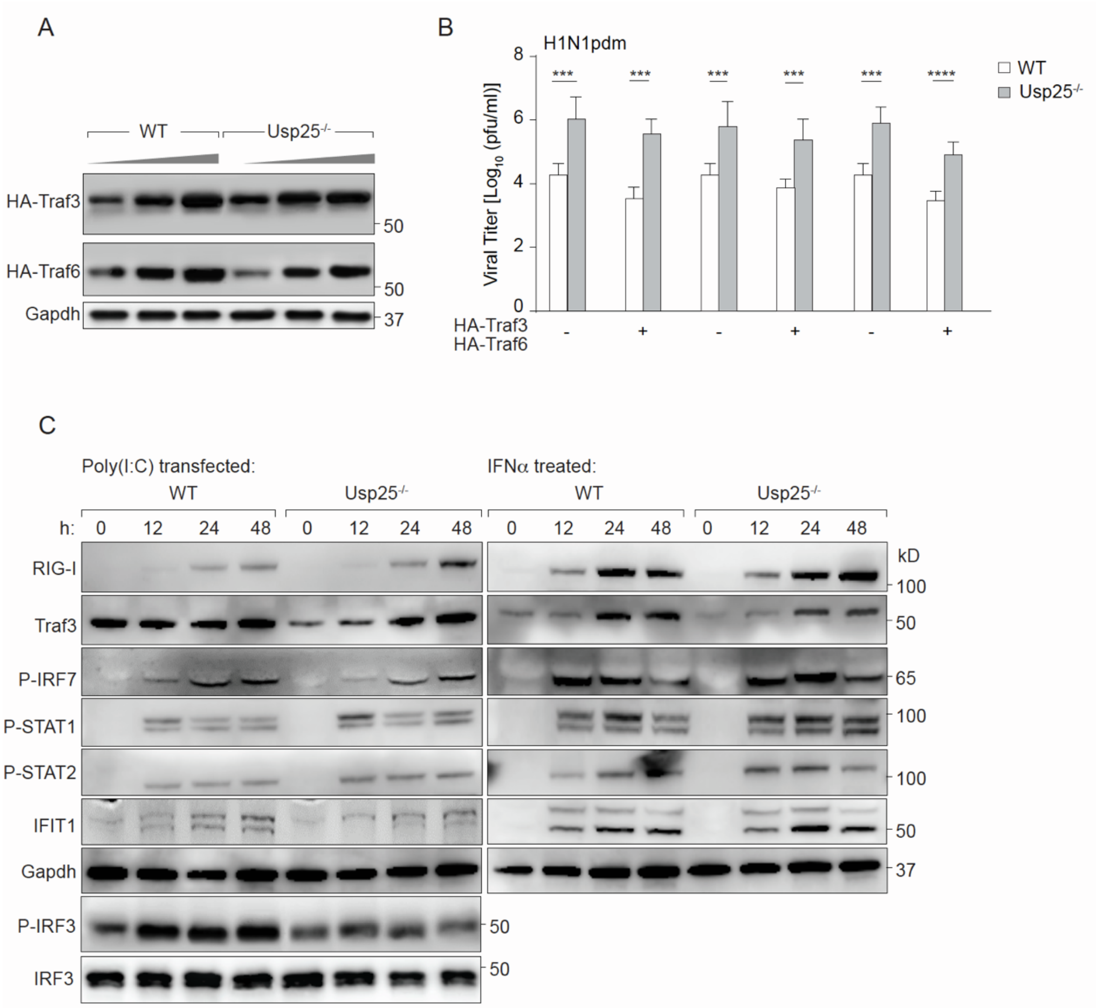

Figure S4. Usp25-deficient cells display attenuated immune responses, related to Figure 4

(**A**) Western blot analyses of Traf3 and Traf6 dose-dependent expression in the WT and Usp25$^{-/-}$ cells. Gapdh was used as loading control. (**B**) Viral titers were measured by plaque assay in cells from (A) infected with H1N1pdm (MOI=0.01) at 24 hours. **(C)** Western blot analysis of RIG-I and downstream effectors of the RIG-I pathway protein expression in WT and Usp25$^{-/-}$ A549 cells treated with either poly I:C or IFN-α at indicated timepoints. Gapdh was measured as loading control.

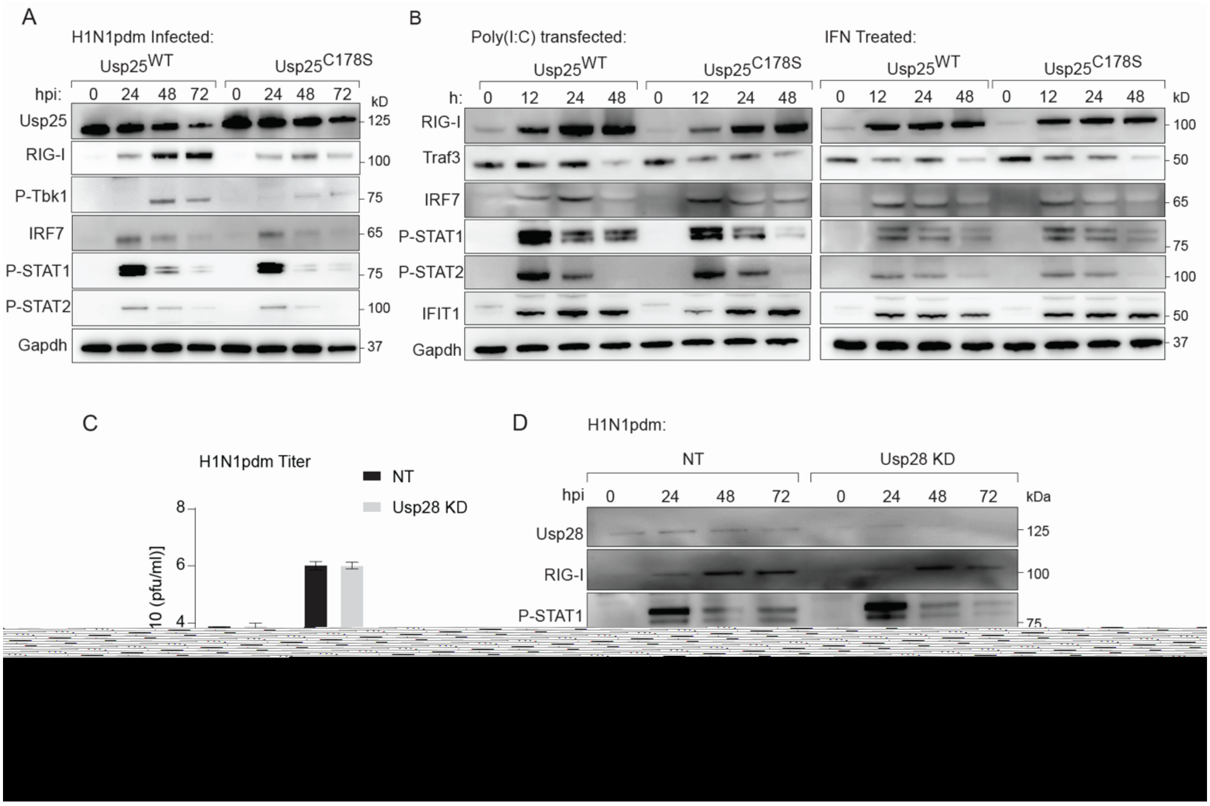

Figure S5. Catalytic activity of Usp25 is necessary for its antiviral function, related to Figure 5

(**A, B**) Western blot analysis of Usp25, RIG-I, and downstream effectors of the RIG-I pathway protein expression in H1N1pdm-infected (A) or either poly I:C or IFN-I treated (B) Usp25^{WT} and Usp25^{C178S} A549 cells. The cells were infected with H1N1pdm at MOI of 0.01. Gapdh was used as loading control.

(**C**) Viral titer of Usp28 knockdown (Usp28 KD) and non-targeting (NT) control A549 cells after H1N1pdm infection at the indicated timepoints (MOI=0.01). Viral titres were determined by plaque assay using MDCK cells. Data are shown as means of n=3 ± standard deviations (SD). Two-way ANOVA was used to analyze data.

(**D**) Western blot analysis of Usp28, RIG-I, phosphorylated STAT1, phosphorylated STAT2 and LC3 protein expression in H1N1pdm-infected NT and Usp28 KD A549 cells. The cells were infected with H1N1pdm (MOI=0.01). Gapdh was used as loading control.

captured on Neutravidin beads followed by re-IP on anti-influenza HA antibodies and detected by autoradiography.

(**F, G**) Western blot analyzes of Rab11, LC3I/II, Lamp2 and Cathepsin B expression in control (shNT) and shErlin1/2 A549 cells at indicated timepoints post H1N1pdm infection (MOI 0.01). Erlin1/2 depleted cells from (F) were treated with DMSO alone or Simvastatin (10μM) prior to H1N1pdm infection (MOI 0.01). Western blotting of proteins was performed in cell lysates prepared at indicated timepoints. Actin levels were measured as a loading control.

(**H**) Control and shErlin1/2 mCherry-GFP-LC3 A549 cells were infected with H1N1pdm (MOI 5). Quantification of autophagosome/autolysosome populations was performed by flow cytometry. Data are shown as mean± SD from 3 independent experiments. [*p<0.1, **p<0.01, ***p<0.001, ****p<0.0001, analyzed by one-way ANOVA].
