# Supplementary material for: Usp25-Erlin1/2 activity limits cholesterol flux to restrict virus infection
Source: Dev Cell. 2023 Nov 20;58(22):2495–2509.e6. doi: 10.1016/j.devcel.2023.08.013 (PMC10914638; doi:10.1016/j.devcel.2023.08.013)
Supplement: Document S2. Article plus supplemental information [file mmc2.pdf]

Article

Developmental Cell

Usp25-Erlin1/2 activity limits cholesterol flux to restrict virus infection

Graphical abstract

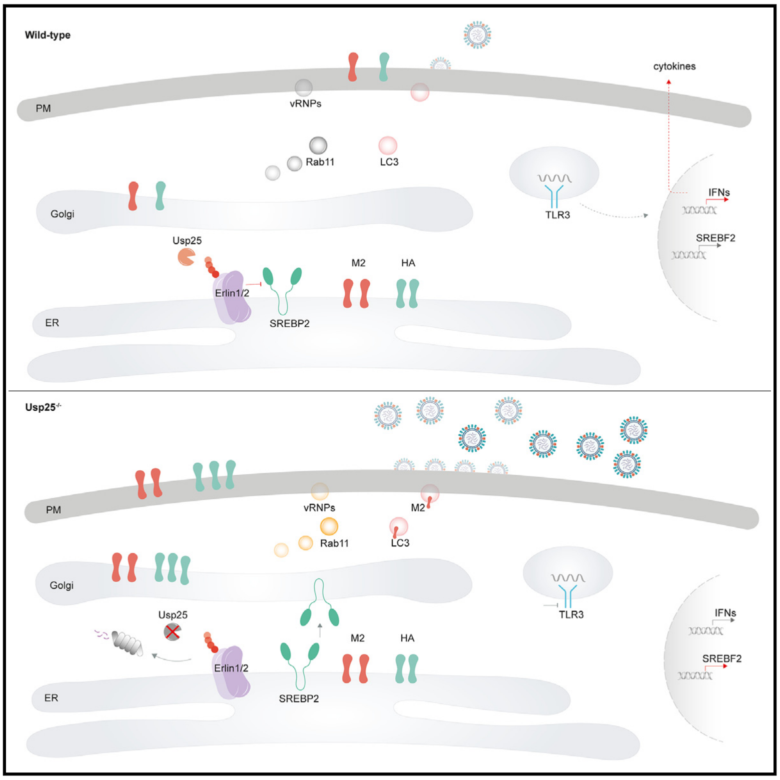

Highlights

- Usp25 deficiency increases susceptibility to pathogenic RNA viruses
- Usp25 stabilizes the Erlin1/2 complex in virus-infected cells
- Erlin1/2 negatively regulates the SREBP2 pathway to block cholesterol synthesis
- Reduced cholesterol levels can trigger TLR3 dependent inflammatory responses

Authors

Qi Wen Teo, Ho Him Wong, Tiaan Heunis, ..., Chris Ka Pun Mok, Rachel Ulferts, Sumana Sanyal

Correspondence

sumana.sanyal@path.ox.ac.uk

In brief

Teo et al. report that Usp25, a deubiquitylase, is able to restrict virus infection by stabilizing the Erlin1/2 complex, which in turn regulates intracellular cholesterol levels. Absence of Usp25 drives Erlin1/2 degradation and increased cholesterol flux, providing a conducive environment for the assembly and secretion of pathogenic viruses.

Teo et al., 2023, Developmental Cell *58*, 2495–2509
November 20, 2023 © 2023 The Author(s). Published by Elsevier Inc.
https://doi.org/10.1016/j.devcel.2023.08.013

Check for updates

CellPress

Developmental Cell

CellPress
OPEN ACCESS

Article

Usp25-Erlin1/2 activity limits cholesterol flux to restrict virus infection

Qi Wen Teo,[1,4] Ho Him Wong,[1] Tiaan Heunis,[2] Viktoriya Stancheva,[2] Asmaa Hachim,[2] Huibin Lv,[1] Lewis Siu,[1] Julian Ho,[1] Yun Lan,[1] Chris Ka Pun Mok,[1] Rachel Ulferts,[3] and Sumana Sanyal[2,1,5,*]

[1]HKU-Pasteur Research Pole, School of Public Health, Li Ka Shing Faculty of Medicine, The University of Hong Kong, Hong Kong SAR, China
[2]Sir William Dunn School of Pathology, South Parks Road, University of Oxford, Oxford OX1 3RE, UK
[3]The Francis Crick Institute, London, UK
[4]Carl R. Woese Institute for Genomic Biology, University of Illinois at Urbana-Champaign, Urbana, IL 61801, USA
[5]Lead contact
*Correspondence: sumana.sanyal@path.ox.ac.uk
https://doi.org/10.1016/j.devcel.2023.08.013

SUMMARY

Reprogramming lipid metabolic pathways is a critical feature of activating immune responses to infection. However, how these reconfigurations occur is poorly understood. Our previous screen to identify cellular deubiquitylases (DUBs) activated during influenza virus infection revealed Usp25 as a prominent hit. Here, we show that Usp25-deleted human lung epithelial A549 cells display a >10-fold increase in pathogenic influenza virus production, which was rescued upon reconstitution with the wild type but not the catalytically deficient (C178S) variant. Proteomic analysis of Usp25 interactors revealed a strong association with Erlin1/2, which we confirmed as its substrate. Newly synthesized Erlin1/2 were degraded in Usp25$^{-/-}$ or Usp25^{C178S} cells, activating Srebp2, with increased cholesterol flux and attenuated TLR3-dependent responses. Our study therefore defines the function of a deubiquitylase that serves to restrict a range of viruses by reprogramming lipid biosynthetic flux to install appropriate inflammatory responses.

INTRODUCTION

Virus outbreaks pose an ongoing risk to public health. Seasonal influenza is estimated to claim approximately 500,000 lives worldwide each year.[1] The coronavirus disease 2019 (COVID-19) pandemic caused by severe acute respiratory syndrome coronavirus 2 (SARS-CoV-2) has resulted in >6 million deaths globally. These virus outbreaks underscore the importance of developing vaccines and antiviral drugs against these pathogens. On the other hand, the rapid evolution of escape mutants that resist host immune responses and virus-directed inhibitors frequently makes developing safe and effective antiviral therapies for influenza and SARS-CoV-2 difficult. Therefore, one approach to responding to an outbreak is to develop drugs or inhibitors against host factors that are critical for the virus life cycle.[2]

Ubiquitylation has been implicated in several critical innate and adaptive immune response processes.[3] Therefore, many viruses, including SARS-CoV and SARS-CoV-2 encode deubiquitylases (DUBs) in order to evade host defenses and establish efficient viral infection.[4,5] Apart from immune evasion, previous studies have shown that ubiquitylation of influenza polymerase basic protein 2 (PB2) and nuclear protein (NP) itself is important for infectious virus particle production.[6,7] Ubiquitylation can, in turn, be hydrolyzed by either virally encoded or cellular DUBs. It is noteworthy that Influenza A viruses (IAVs) do not express their own DUBs. We previously employed activity-based protein profiling to identify DUBs activated during IAV infection and immune signaling.[8,9] Among others, we identified Usp25, which was activated in an infection-specific manner.

In vivo studies in Usp25$^{-/-}$ mice have indicated that Usp25 inhibits IL-17-triggered signaling,[10] and activates interferon production by stabilizing Traf3 and Traf6.[11] However, the cell-intrinsic mechanism of Usp25 function is less clear.

Here, we identify a critical role of Usp25 in metabolic reprogramming upon challenge with influenza and SARS-CoV-2. Usp25$^{-/-}$ cells display >10-fold increase in susceptibility to both viruses, with concomitant decreases in interferon and proinflammatory cytokine production. The Usp25 interactome revealed its strongest association with Erlin1/2, which, in turn, regulated Srebp2 activation. Reconstituting the wild-type but not the catalytically inactive variant in the Usp25$^{-/-}$ cells rescued this phenotype. The Usp25-deficient phenotype could be recapitulated in Erlin1/2-depleted cells. Our data show that Usp25-Erlin1/2 activity regulates cholesterol biosynthesis and efflux in response to TLR3-dependent sensing of viral RNA. Although ubiquitin-dependent immune responses have been widely reported, their regulation of immunometabolic processes has been far less studied. Our results therefore define a previously uncharacterized Usp25-regulated pathway for cholesterol biosynthetic flux to restrict RNA virus infections.

© 2023 The Author(s). Published by Elsevier Inc.
This is an open access article under the CC BY license (http://creativecommons.org/licenses/by/4.0/).

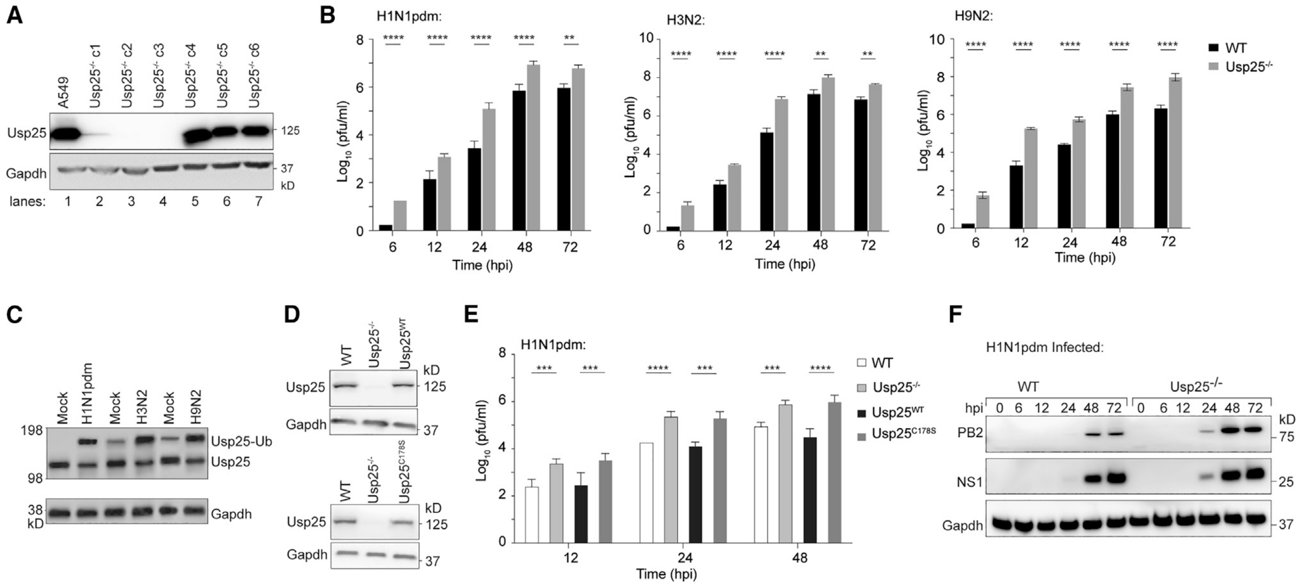

Figure 1. Usp25 deficiency enhances group 1 and group 2 influenza virus production

(A) Western blots validating CRISPR/Cas9-mediated Usp25 deletion (Usp25$^{-/-}$) in A549 cells. Gapdh expression was measured as loading control.

(B) Viral titer of wild-type (WT) and Usp25$^{-/-}$ A549 cell was measured in H1N1pdm, H3N2, and H9N2 influenza infection at the indicated time points (MOI = 0.01). Viral titers were determined by plaque assay using MDCK cells. Data are shown as means of n = 3 ± standard deviations (SD). Two-way ANOVA was used to analyze data; **$p < 0.01$, ****$p < 0.0001$. * denotes significant difference of viral titer between cells.

(C) HA-Ub-VME (10 μM) was added to permeabilized control cells or those infected with indicated viruses. Lysates generated were resolved by SDS-PAGE and immunoblotted with anti-Usp25.

(D) Western blots validating WT Usp25 (Usp25^{WT}) and catalytically inactive mutant of Usp25 (C178S) (Usp25^{C178S}) rescued A549 cells. Gapdh was measured as loading control.

(E) Viral titers of WT, Usp25$^{-/-}$ cells, Usp25^{WT}, and USP25^{C178S} A549 cells after H1N1pdm infection at the indicated timepoints (MOI = 0.01), determined by plaque assay using MDCK cells. Data are shown as means of n = 3 ± standard deviations (SD). Data analyzed by two-way ANOVA (***$p < 0.001$, ****$p < 0.0001$).

(F) Western blot analysis of PB2 and NS1 protein expression upon H1N1pdm infection in WT and Usp25$^{-/-}$ A549 cells at the indicated time points (MOI = 0.01). Gapdh was used as loading control.

See also Figure S1.

RESULTS

Usp25$^{-/-}$ cells display increased susceptibility to virus infection

Usp25 emerged as a DUB that is activated in virus-infected cells.[9] In order to investigate the function of Usp25 during viral infection, we first generated Usp25$^{-/-}$ cells using CRISPR/Cas9 technology. The experiments described in the study were conducted using two separate Usp25$^{-/-}$ clones (c2 and c3) (Figure 1A). Because hemagglutinin (HA) subtypes of IAVs can be clustered into group 1 (e.g., H1 and H5) and group 2 (e.g., H3 and H7), we challenged control and Usp25$^{-/-}$ cells with group 1 H1N1pdm, group 2 H3N2 and avian flu H9N2 at multiplicity of infection (MOI) of 0.01, as well as with SARS-CoV-2 and Zika viruses. Supernatants were collected from the infected cells at 0, 6, 12, 24, 48, and 72 h post infection (hpi) and viral titers were measured by plaque assay. Virus production from Usp25$^{-/-}$ cells was 1.5–2 logs higher than the wild-type cells for all IAV strains and SARS-CoV-2 (Figures 1B and S1A–S1C) and also from primary epithelial cells (Figure S1D). For all IAV strains, the fraction of Usp25 captured by HA-Ub-vinylmethylester (VME), a DUB-specific activity-based probe, is significantly higher in virus-infected cells compared with mock-infected cells (Figure 1C). In addition, we generated A549 cells stably expressing either wild-type (Usp25^{WT}) or the catalytically dead variant (Usp25^{C178S}) in the Usp25$^{-/-}$ background. The cysteine residue 178 (Cys178) is essential for Usp25 function, and mutation results in complete loss of its catalytic activity.[12] We reconstituted both the wild-type Usp25 and catalytically inactive mutant of Usp25 (C178S) into the Usp25$^{-/-}$ cells, which expressed at similar levels (Figure 1D). Although the expression of Usp25^{WT} attenuated virus production, the expression of Usp25^{C178S} did not have any effect, suggesting that the catalytic activity of Usp25 is necessary for this phenotype (Figure 1E). In line with increased virus production, lysates from infected Usp25$^{-/-}$ cells had increased expression of viral proteins compared with that of wild-type cells (Figure 1F). Both PB2 and NS1 were detectable at 24 hpi in Usp25$^{-/-}$ cells but not in wild-type cells. A modest decrease in Usp25 expression occurred over the course of infection (Figures S1E and S1F).

To further determine the specific stage in the viral life cycle regulated by Usp25, we first assessed viral entry. Wild-type and Usp25$^{-/-}$ cells were infected with R18-labeled IAV for 1 h. The membrane-associated fluorescence of R18 is self-quenched at high concentrations but becomes detectable upon dilution and can be used to quantify viral entry and fusion with the endosomal membrane (Figure S1G). In parallel, we also quantified NP-positive cells at early time points in infection

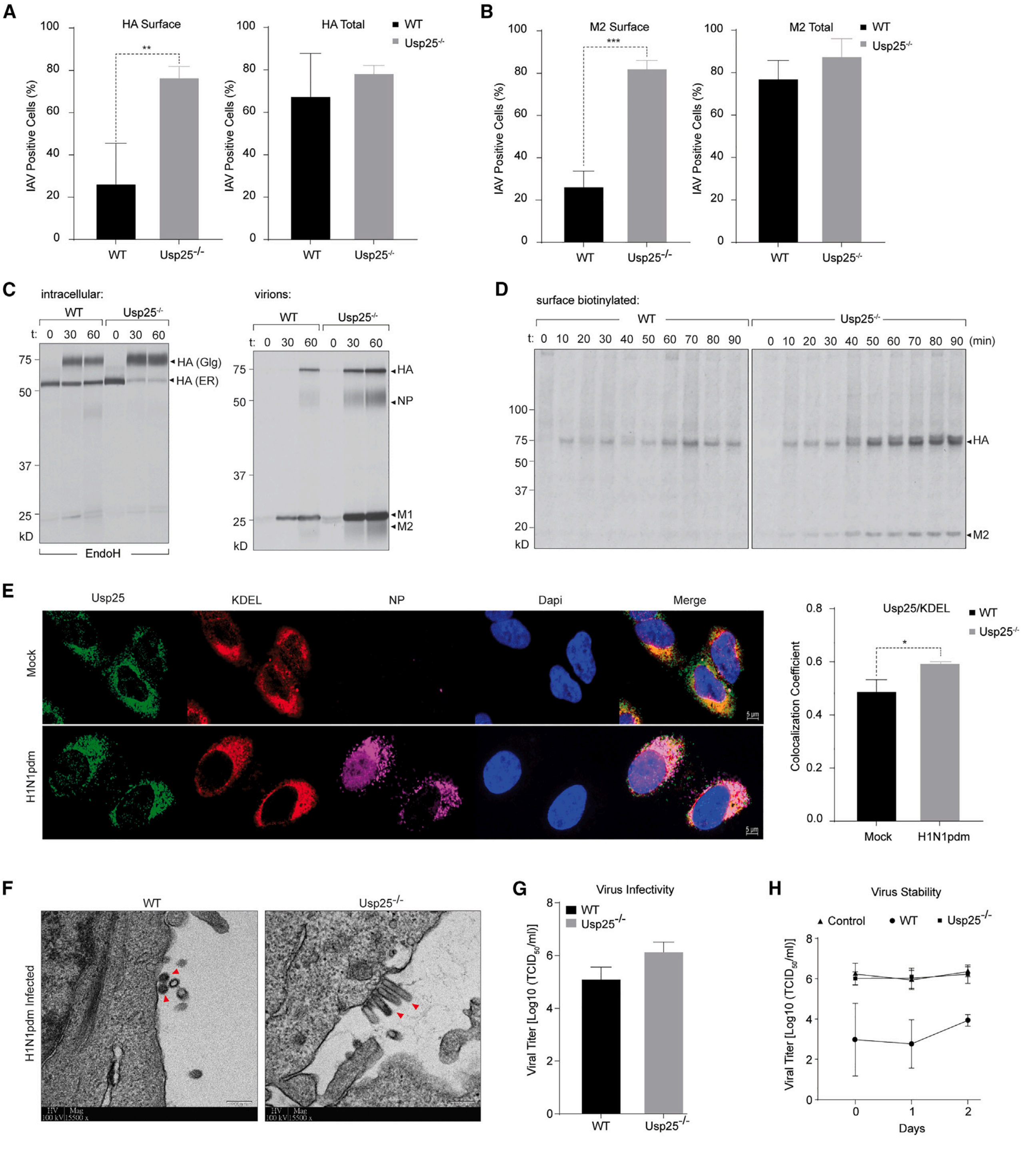

Figure 2. Usp25 restricts late stages in virus infection

(A and B) WT and Usp25$^{-/-}$ A549 cells were infected with H1N1pdm virus and total versus surface expression of viral HA and M2 were quantified by flow cytometry (MOI = 5). Data are shown as means of n = 3 ± standard deviations (SD). Data analyzed by Student's unpaired t test was (**$p < 0.01$, ***$p < 0.001$).

(C, left panel) intracellular trafficking of viral HA was measured in IAV-infected (MOI = 1) wild-type or Usp25$^{-/-}$ A549 cells. 10 hpi cells were pulsed with [^{35}S] cysteine/methionine for 10 min, followed by chase in complete media for 30 and 60 min. HA was immunoprecipitated, Endo H-treated, resolved by gel electrophoresis, and detected by autoradiography. (Right panel) Released virions were concentrated on chicken erythrocytes, resolved on SDS-PAGE, and detected by autoradiography.

(D) [^{35}S]cysteine/methionine-labeled cells were chased for indicated time points; surface biotinylation was performed using NHS-SS-biotin for 1 h at 4°C. Biotinylated proteins were captured on streptavidin beads, cleaved using DTT, and immunoprecipitated with anti-HA antibodies.

(legend continued on next page)

(Figure S1H). Entry and fusion remained unaffected in Usp25$^{-/-}$ cells. To measure replication, we infected wild-type and Usp25$^{-/-}$ cells with IAV (H1N1pdm) at a MOI of 5 for a single-cycle viral infection. At 2, 4, 6, 8, and 10 hpi, total cellular RNA was extracted, and virus M gene copy numbers of vRNA, cRNA, and mRNA were measured using RT-qPCR, which confirmed that viral replication was not affected in Usp25$^{-/-}$ cells (Figures S1I and S1J). In addition, Usp25 did not associate with the viral proteins PB2, NP, and NS1 as tested via co-immunoprecipitations (Figures S1K and S1L). Collectively, these data indicate that Usp25 does not regulate viral entry or replication but most likely affects later stages to inhibit the production of infectious progeny virions.

Usp25 regulates trafficking and secretion of IAV

To test the role of Usp25 in the later stages of the viral life cycle, we examined trafficking of viral structural proteins to the cell surface. The level of haemagglutinin (HA) and M2 at the cell surface was measured in wild-type and Usp25$^{-/-}$ cells infected with IAV at MOI 5, 8 hpi using flow cytometry (Figures 2A and 2B). Cells were either permeabilized to assess the total intracellular levels or left intact to assess only the cell surface pool of viral HA and M2. Wild-type and Usp25$^{-/-}$ cells displayed similar levels of total HA and M2; however, surface expression of both in the Usp25$^{-/-}$ cells was 30%–40% more than in the wild-type cells (Figures 2A and 2B), indicating faster kinetics of HA and M2 trafficking in the Usp25$^{-/-}$ cells compared with wild-type control cells. Transport via the secretory pathway was also verified using brefeldin A treatment, which significantly reduced surface expression in both wild-type and Usp25$^{-/-}$ cells (Figure S2A).

To verify the phenomenon of increased trafficking of newly synthesized HA and M2, we performed pulse-chase assays by radioactive labeling coupled with surface biotinylation. HA is synthesized at the rough endoplasmic reticulum (ER) and glycosylated at the ER and Golgi before arriving at the apical cell surface.[13] To measure HA trafficking, we radiolabeled IAV-infected wild-type and Usp25$^{-/-}$ cells with [^{35}S]cysteine/methionine for 10 min and chased for indicated time intervals in cold media (Figures 2C and 2D). At each time point, HA was immunoprecipitated and treated with EndoglycosidaseH to measure the ER versus Golgi/PM-resident fractions. In line with the FACS data, trafficking of HA in Usp25$^{-/-}$ cells occurred with significantly faster kinetics compared with that in wild-type cells (Figure 2C), with a subsequent increase in the secretion of virus progenies (Figure 2C). We biotinylated the cell surface fraction of HA and M2 using cleavable NHS-SS-biotin. Biotinylated proteins were first immunoprecipitated on streptavidin beads from wild-type and Usp25$^{-/-}$ cells infected with IAV and pulse labeled with [^{35}S]cysteine/methionine. Eluted material was further immunoprecipitated on specific antibodies against HA. Expression of both HA and M2 at the cell surface was detected to be significantly higher in Usp25$^{-/-}$ cells compared with the wild-type (Figure 2D). To determine whether Usp25 was recruited to the secretory pathway, we measured its colocalization with the KDEL receptor in mock and IAV-infected cells, which revealed a modest increase in the infected cells (Figure 2E).

To visualize the final stages in the IAV life cycle, we performed EM-imaging of wild-type and Usp25$^{-/-}$ cells infected with IAV. Although we detected spherical IAV progenies budding from the surface of wild-type cells, this phenotype was altered in Usp25$^{-/-}$ cells, where progeny virions displayed a filamentous morphology (Figures 2F, S2B, and S2C). Infectivity and stability of IAV progenies measured from wild-type and Usp25$^{-/-}$ cells did not display significant differences (Figures 2G and 2H).

The morphology of IAV is primarily driven by the viral M2 protein.[14] In particular, interaction of viral M2 with cholesterol was reported to drive filamentous virions.[15] We therefore aimed to determine whether Usp25 regulated the expression and ubiquitylation of M2. Surprisingly, increasing expression levels of wild-type (Usp25^{WT}) but not catalytically deficient Usp25 (Usp25^{C178S}) resulted in a dose-dependent decrease in viral M2 levels (Figure S2D). These data indicate that M2 is not a direct substrate of Usp25 because activity of Usp25^{WT} is expected to stabilize its potential substrates; however, Usp25 activity very likely triggers M2 turnover via recruitment of E3-ligases followed by either lysosomal or proteasomal degradation. In addition, cells co-expressing M2 with either Usp25^{WT} or Usp25^{C178S} revealed that although typical and atypical ubiquitin chains could be isolated on M2, Usp25-dependent hydrolysis occurred specifically for K6- and K63-linked ubiquitin chains (Figures S2E and S2F). This could potentially lead to increased K48-linked ubiquitylation and degradation. Collectively, these data indicate that Usp25 inhibits the later stages of the viral life cycle, particularly the expression and trafficking of viral structural proteins.

Inhibition of autophagic flux in IAV-infected Usp25$^{-/-}$ cells

One of the pathways routinely targeted by a range of viruses is autophagy.[16–20] The later stages of the IAV life (transport and assembly) are facilitated by LC3+ compartments (for M2 transport)[21] and Rab11 (for vRNP transport).[22] In particular, M2 of IAV was shown to interact with LC3 and redistribute these compartments to the plasma membrane for virus budding.[21] Recent studies indicate that ubiquitylation of M2 by the MARCH8 E3-ligase redirects it for lysosomal degradation.[23] Both LC3 and Rab11 expression levels were significantly enhanced in both mock and virus-infected Usp25$^{-/-}$ cells compared with the wild type (Figures 3A–3C). These data indicate that deletion

(E) Immunofluorescence imaging of Usp25 and KDELR were performed in mock and IAV-infected cells (MOI = 5). Colocalization coefficient between mock and IAV-infected A549 cells was measured using Student's unpaired t test (*p < 0.1).
(F) Negative-stain EM was performed in thin sections from IAV-infected wild-type and Usp25$^{-/-}$ cells. Red arrows indicate budding virions.
(G) A549 cells were infected with cell-free virus particles collected from wild-type and Usp25$^{-/-}$ A549 cells (MOI = 0.01). Viral titers were determined by 50% tissue culture infectious dose (TCID$_{50}$/ml) using MDCK cells. Data are shown as means of n = 3 ± standard deviations (SD). Data analyzed by Student's unpaired t test.
(H) Viral stock (control) and cell-free supernatant collected from wild-type and Usp25$^{-/-}$ A549 cells were frozen either immediately or after having been left at room temperature for indicated times. Viral titers were determined by TCID$_{50}$/ml using MDCK cells. Data are shown as means of n = 3 ± standard deviations (SD). Data analyzed by two-way ANOVA.
See also Figure S2.

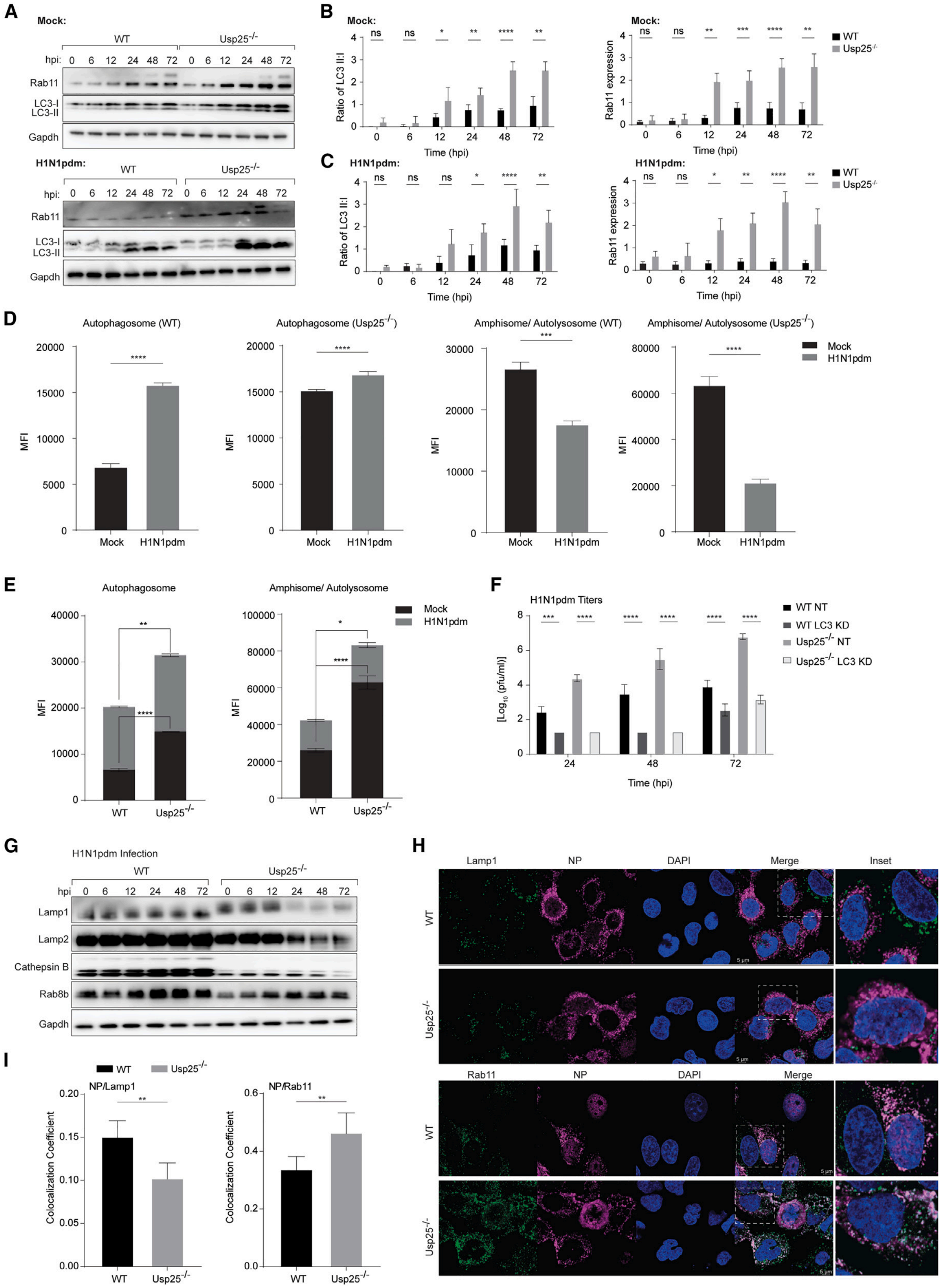

Figure 3. Usp25 deletion increases autophagosome abundance

(A) Western blot analysis of LC3-I/II and Rab11 protein expression in WT and Usp25$^{-/-}$ A549 cells at indicated time points. Cells were either mock-infected or H1N1pdm-infected at MOI of 0.01. Gapdh levels were measured as a loading control.

(legend continued on next page)

of Usp25 results in either decreased autophagic flux or specific induction of non-canonical LC3+ compartments previously described to assist in viral protein transport.[21]

To distinguish between degradative versus non-classical LC3+ compartments, we generated a stable reporter cell line with fluorescently tagged LC3 (mCherry-GFP-LC3) and measured autophagic flux using flow cytometry.[16,17] The principle of this assay is based on the pH resistance of mCherry versus the sensitivity of GFP fluorophores to the acidic environment of late endosomes and lysosomes. In line with the immunoblots, quantification by flow cytometry indicated accumulation of autophagosomes upon infection in both wild-type and Usp25$^{-/-}$ cells, with a significantly higher population of non-degradative autophagosomes in the Usp25$^{-/-}$ cells for both mock and infected cells (Figures 3D, 3E, and S3A). We hypothesized that these LC3+ compartments in Usp25$^{-/-}$ cells provide a cellular environment that primes the cell to favor viral M2-dependent IAV transport and budding, resulting in higher viral production observed as early as 6 hpi in the Usp25$^{-/-}$ cells. In contrast, the abundance of amphisomes and/or autolysosomes was reduced upon infection, more significantly in the Usp25$^{-/-}$ cells, suggesting that IAV inhibits the degradative arm of the autophagosomal pathway to prevent virus clearance (Figures 3D and 3E).

To test whether other components of the autophagosomal pathway were also altered, we measured expression levels of Beclin-1 (a conventional autophagy marker), ATG5, and ATG12 in IAV-infected wild-type and Usp25$^{-/-}$ cells, and none of them displayed detectable differences between wild-type and Usp25$^{-/-}$ cells (Figure S3B). However, when we measured ULK1, an early autophagy marker, by confocal imaging, a significant increase in punctate structures was detected in the Usp25$^{-/-}$ cells compared with wild type, in line with the increase in LC3-II compartments (Figure S3C). To specifically test the role of LC3 in IAV production, we generated both wild-type and Usp25$^{-/-}$ cells that were depleted in LC3 (Figure S3D). In both cases, LC3-depleted cells displayed dramatically reduced IAV titers, underscoring the key role it plays in the IAV life cycle (Figure 3F). In contrast, depletion of Beclin-1 did not have any detectable effect on virus production (Figure S3E).

To further characterize autolysosomal inhibition, we measured the steady-state levels of lysosomal markers in wild-type and Usp25$^{-/-}$ IAV-infected cells. Expression of Lamp1 and Lamp2 decreased significantly at 24 hpi in the Usp25$^{-/-}$ cells compared with that in the wild type, reflecting a decrease in the number of functional lysosomes (Figure 3G). In addition, we observed a marked reduction in both the precursor and mature forms of cathepsin B (lysosomal hydrolase) in the Usp25$^{-/-}$ cells, along with Rab8b, known to be involved in degradative autophagy[24,25] (Figure 3G). The decrease in Lamp1 and increase in Rab11 abundance in IAV-infected cells was also observed by immunofluorescence imaging (Figures 3H and 3I). These data are in line with our observation that autophagic degradation is arrested in the Usp25$^{-/-}$ cells. Interestingly, Usp25 deletion resulted in Lamp1 hyperglycosylation (Figure S3F), which is linked to lysosomal dysfunction[26] and altered cellular cholesterol homeostasis.[27] This phenomenon was also evident in [^{35}S]cysteine/methionine-labeled cells, particularly in the Usp25^{C178S} catalytically dead variant-expressing cells (Figure S3G). Collectively, these data indicate that (1) the non-degradative LC3+ population is increased in the Usp25$^{-/-}$ cells to promote virus release and (2) both autophagic degradation and lysosomal activity in Usp25$^{-/-}$ cells are suppressed, which together facilitate virus production from these cells.

Usp25 deficiency suppresses innate immune signaling in IAV-infected cells

Because Usp25 inhibited late stages in the viral life cycle, we aimed to determine if this occurred as a consequence of increased innate immune responses to infection. Previous reports indicated that Usp25 regulates the expression of Traf3 and Traf6.[11] Therefore, to test whether the effect of increased IAV infection was on account of Traf3 and Traf6 degradation, we co-expressed Traf3 and Traf6 in the wild-type and Usp25$^{-/-}$ cells. Although expression of Traf3 and Traf6 moderately suppressed infection, they did not alter the increase in IAV production from Usp25$^{-/-}$ cells (Figures S4A and S4B). IAV infection in A549 cells triggers TLR3, RIG-I, and IFN-I signaling pathways.[28] To test the effect of Usp25 on these pathways, we first measured the expression profiles of common immune effectors. Compared with the wild-type cells, Usp25$^{-/-}$ cells showed a significant reduction in RIG-I protein expression during the course of IAV infection (Figure 4A). Downstream effectors such as Traf3, pTbk1, pIRF3, and IRF7, as well as IFNAR effectors, were mildly attenuated (Figure 4A). Secretion of several proinflammatory cytokines such as IL-6, TNFα, IP-10 (CXCL10), and IFN-λ1 was substantially reduced (Figure 4B). In contrast, secretion of chemokines such as RANTES, MIP-3α (CCL20), MIP-1β (CCL4), and I-TAC (CXCL11) remained unaltered or moderately upregulated in the Usp25$^{-/-}$ cells upon infection (Figure 4C).

(B and C) Ratio of LC3-II to LC3-I and Rab11 expression normalized to Gapdh were quantified by densitometry. Data are shown as means of n = 3 ± standard deviations (SD). Data analyzed by two-way ANOVA (*p < 0.1, **p < 0.01, ***p < 0.001, ****p < 0.0001; ns: not significant).

(D and E) WT and Usp25$^{-/-}$ mCherry-GFP-LC3 stable A549 cells were infected with H1N1pdm at MOI = 5. Quantification of the populations in the cells were performed by flow cytometry. Data are shown as means of n = 3 ± standard deviations (SD). Data analyzed by Student's unpaired t test (*p < 0.1, **p < 0.01, ***p < 0.001, ****p < 0.0001).

(F) siRNA-mediated depletion of LC3 was performed in WT and Usp25$^{-/-}$ A549 cells followed by infection with H1N1pdm strain (MOI = 0.01). At indicated time points, viral titers were measured by plaque assay. Data are shown as means of n = 3 ± standard deviations (SD). Two-way ANOVA was used to analyze data (***p < 0.01, ****p < 0.0001).

(G) Western blot analysis of Lamp1, Lamp2, cathepsin B, and Rab8b protein expression in WT and Usp25$^{-/-}$ A549 cells after H1N1pdm infection at indicated time points (MOI = 0.01). Gapdh was measured as loading control.

(H) Immunofluorescence images of A549 cells infected with H1N1pdm viruses at MOI = 5 was shown. Viral protein (NP) is depicted in magenta; Lamp1, Rab11 are in green.

(I) Colocalization of NP with Lamp1 and Rab11 between WT and Usp25$^{-/-}$ A549 cells quantified by Student's unpaired t test (**p < 0.01).

See also Figure S3.

CellPress
OPEN ACCESS

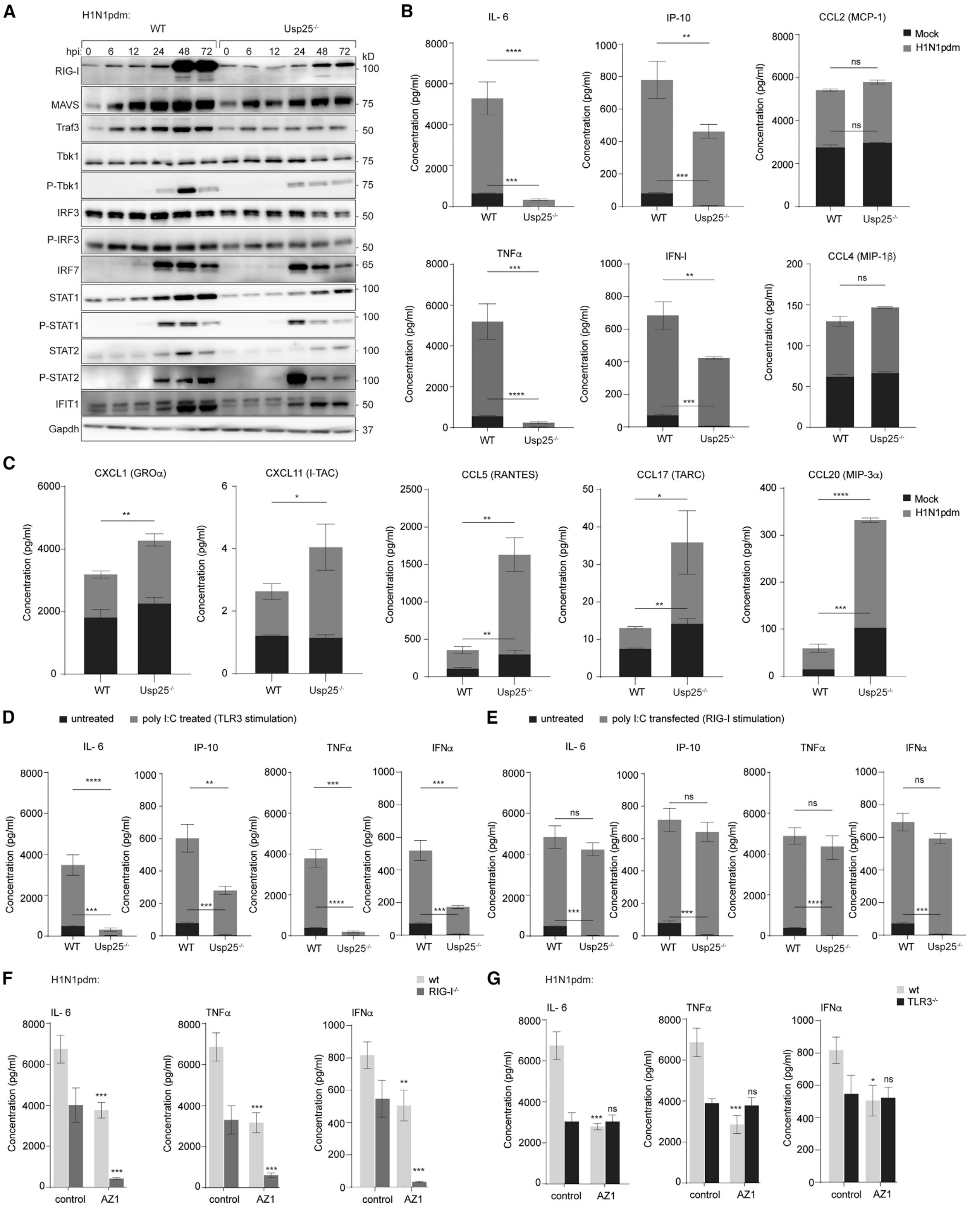

Figure 4. Usp25-deficiency dampens TLR3-dependent inflammatory responses

(A) Western blot analysis of RIG-I and downstream effectors of the RIG-I pathway protein expression in wild-type and Usp25$^{-/-}$ A549 cells after H1N1pdm infection at indicated time points (MOI = 0.01). Gapdh was measured as loading control.

(legend continued on next page)

To decouple virus-triggered immune suppression from Usp25-dependent immune dysregulation, we stimulated wild-type and Usp25$^{-/-}$ cells with transfected poly (I:C) or exogenous IFNα as a means to trigger RIG-I (via poly [I:C]) or IFNAR (via IFNα) (Figure S4C). Interestingly, no significant change in RIG-I expression or its downstream effectors was detected between wild-type and Usp25$^{-/-}$ cells (Figure S4C). These data indicate that RIG-I suppression is not directly due to Usp25 deficiency but rather a virus-triggered effect. However, suppression of proinflammatory cytokine production remained significantly low in Usp25$^{-/-}$ cells specifically stimulated with extracellular poly I:C but not when transfected, suggesting defective TLR3 signaling and not RIG-I being attenuated in Usp25$^{-/-}$ cells (Figures 4D and 4E).

To further distinguish RIG-I versus TLR3-dependent immune suppression in Usp25-deficient cells, we inhibited Usp25 using AZ1, a specific dual inhibitor of Usp25/Usp28 in wild-type, RIG-I$^{-/-}$, or TLR3$^{-/-}$ cells.[29] Cells were then infected with H1N1pdm to measure the selected cytokines. Pharmacological inhibition of Usp25 in RIG-I$^{-/-}$ but not TLR3$^{-/-}$ cells abolished cytokine production, indicating attenuation of cytokine responses in Usp25$^{-/-}$ cells was TLR3 mediated (Figures 4F and 4G).

The catalytic activity of Usp25 is necessary for its antiviral function

Given that reconstitution with wild-type Usp25 (Usp25^{WT}) but not the catalytically inactive mutant (Usp25^{C178S}) restored viral titers (Figure 1C), we aimed to test whether the increased abundance of autophagosomal compartments and suppressed immune responses in Usp25$^{-/-}$ cells are recapitulated with Usp25^{C178S}. Usp25$^{-/-}$ A549 cells were transduced with Usp25^{WT} or Usp25^{C178S} for stable expression and infected with H1N1pdm. Expression of LC3-I/II, Rab11, and Lamp1 was measured by immunoblotting at each time point. In line with results from wild-type control and Usp25$^{-/-}$ cells, abundance of LC3 and Rab11 compartments was increased in Usp25^{C178S} but not in Usp25^{WT} cells (Figures 5A and 5B). As observed for Usp25$^{-/-}$ cells, proinflammatory cytokine responses were attenuated in virus-infected and poly (I:C) stimulated Usp25^{C178S} but not in Usp25^{WT} cells (Figures 5C and 5D). Similarly, RIG-I expression was suppressed upon IAV infection but not upon poly (I:C) transfection or IFN-I treatment, confirming this feature as virus-mediated rather than Usp25-dependent (Figures S5A and S5B). Collectively, these data indicate that the catalytic activity of Usp25 is necessary for restricting LC3 and Rab11 compartments and IAV production.

Usp28 is a close homolog of Usp25 with structural similarities.[30] To elucidate whether the Usp25-dependent phenotype in IAV infection is a specific feature of Usp25, we depleted Usp28 using siRNA and subjected the Usp28-deficient cells to IAV infection. In contrast to the effect of Usp25 deletion, our results showed that viral titers, RIG-I signaling, and autophagy were not affected upon Usp28 depletion, indicating that the antiviral function of Usp25 is specific (Figures S5C and S5D). The distinct results displayed by Usp25 and Usp28 during IAV infection may well be due to different subcellular localizations; Usp28 resides in the nucleus, whereas Usp25 can be found both in the nucleus and cytoplasm.[31] Although Usp25 and Usp28 share a high degree of sequence identity and domain structure, their activity is regulated by distinct oligomerization states.[31] In line with these findings, our results indicate that, in the context of infection, the antiviral role of Usp25 is specific.

Usp25 interacts with and stabilizes the Erlin1/2 complex

To determine Usp25 interactors in mock and IAV-infected cells, we immunoprecipitated proteins from cells expressing FLAG-tagged Usp25. Immunoprecipitated material from mock and IAV-infected cells were subjected to trypsin digestion and analyzed by mass spectrometry. Proteomic analyses revealed that Usp25 interacted with Erlin1 and Erlin2 in both mock and IAV-infected cells (Figure 6A). The Erlin1/2 complex is involved in ER-associated degradation. In addition, Usp25 interacted with several proteins specifically in IAV-infected cells (Figure 6B). In particular, IAV-infected cells displayed an enrichment of the mitochondrial protein complex Tufm-Nlrx1 that has previously been implicated in autophagy, and RIG-I signaling immune checkpoint[32] and RALY (a regulator of LXR), which is a transcriptional regulator of cholesterol efflux.[33] The viral M2 and NP proteins were also found to co-precipitate with Usp25 from IAV-infected cells.

To confirm the role of Usp25 in Erlin1/2, we first measured their expression profiles in mock and virus-infected cells. Erlin1/2 expression was found to increase upon Usp25 expression and decrease in Usp25$^{-/-}$ or Usp25^{C178S} cells (Figure 6C). To confirm whether Erlin1/2 were Usp25 substrates, we performed pulse-chase assays in cells radiolabeled with [^{35}S]cysteine/methionine. In both the Usp25$^{-/-}$ and Usp25^{C178S} cells, Erlin1 and 2 were found to undergo rapid turnover, with higher molecular weight forms similar to ubiquitylation apparent in the Usp25^{C178S} cells. On the other hand, Erlin1/2 levels were stabilized in the Usp25^{WT} cells (Figures 6D and 6E), indicating that turnover of the Erlin1/2 complex might be regulated by Usp25.

To validate the IAV-specific interactions identified by mass spectrometry, we co-transfected Myc-Usp25 with FLAG-tagged constructs in HEK293T cells (Figure 6F). We could detect interaction of Usp25 with IFIT1, IFIT5 and viral M2 (both wild type and a LC3-interaction region mutant version M2F91S). Interaction of Erlin1/2 with Usp25 was validated using a BioID construct

(B and C) Cell culture supernatants in mock and H1N1pdm infected WT and Usp25$^{-/-}$ A549 cells were harvested (MOI = 0.01), and cytokine/chemokine profiles were determined by flow cytometry using the LEGENDplex antiviral and proinflammatory cytokine response panel. Data are shown as means of n = 3 ± standard deviations (SD). Data analyzed by Student's unpaired t test (*p < 0.1, **p < 0.01, ***p < 0.001, ****p < 0.0001; ns: not significant).
(D and E) WT and Usp25$^{-/-}$ cells were either stimulated with 25 μg/ml poly (I:C) and added to cell culture for 24 h to trigger TLR3 or transfected with 10 μg/ml poly (I:C) with Lipofectamine to trigger RIG-I for 24 h. Cytokine profiles were determined as described in (B). (*p < 0.1, **p < 0.01, ***p < 0.001, ****p < 0.0001; ns: not significant as analyzed by Student's unpaired t test).
(F and G) WT, RIG-I$^{-/-}$ and TLR3$^{-/-}$ cells were infected with H1N1pdm (MOI 0.01, 24 h) in the presence of AZ1 (5 μM). Cytokine profiles were determined as described in (B). (*p < 0.1, **p < 0.01, ***p < 0.001, ****p < 0.0001; ns: not significant as analyzed by Student's unpaired t test).
See also Figure S4.

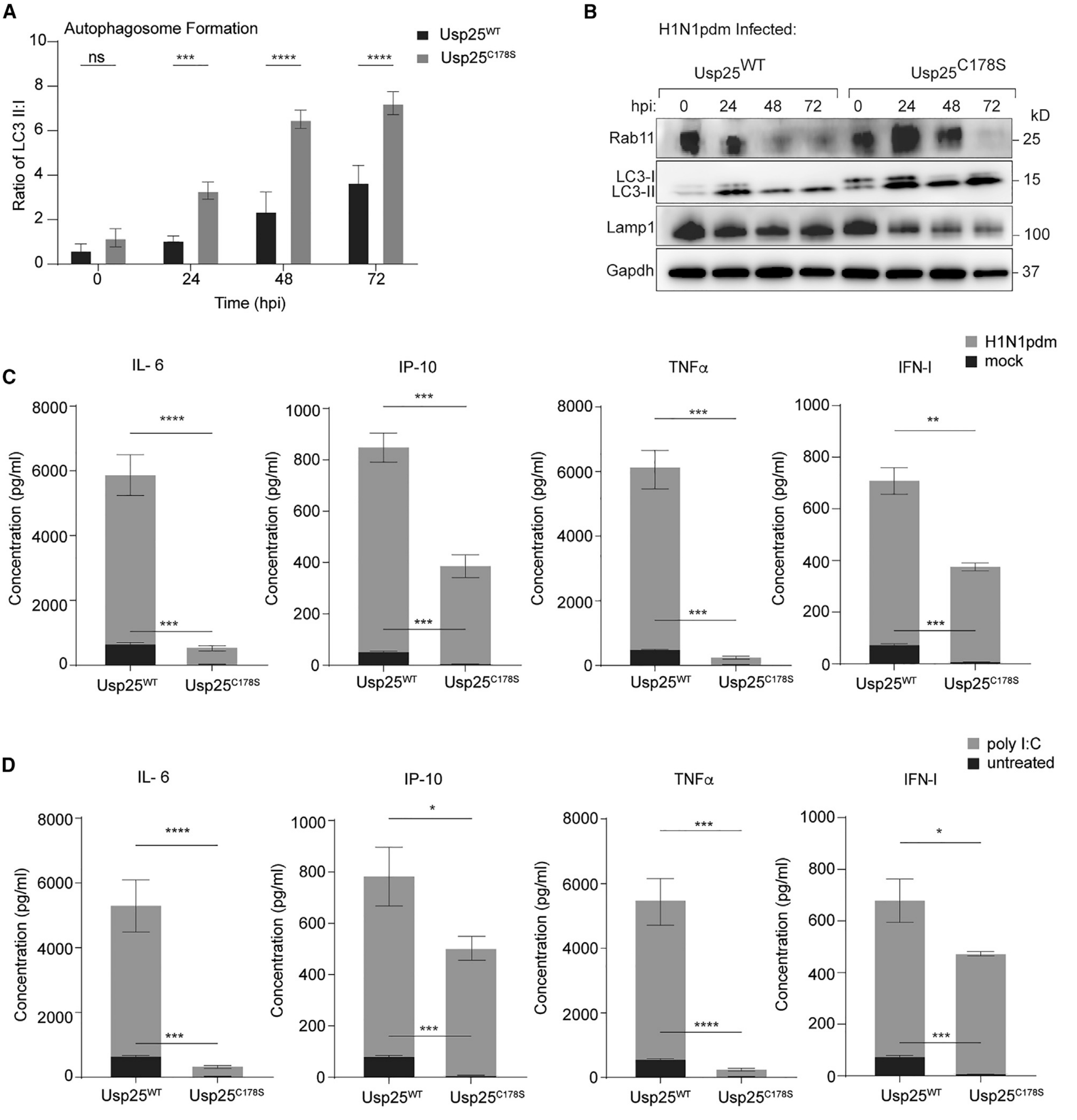

Figure 5. Catalytically deficient Usp25 recapitulates the Usp25$^{-/-}$ phenotype in virus infection

(A) Ratio of LC3-II to LC3-I used to measure LC3-II accumulation in H1N1pdm-infected Usp25^{WT} and Usp25^{C178S} A549 cells (MOI = 0.01). Data are shown as mean of n = 3 ± standard deviations (SD). Data analyzed by two-way ANOVA (***p < 0.001, ****p < 0.0001; ns: not significant).

(B) Western blot analysis of Rab11, LC3, and Lamp1 protein expression in H1N1pdm-infected Usp25^{WT} and Usp25^{C178S} A549 cells (MOI = 0.01). Gapdh levels measured as a loading control.

(C and D) Cell culture supernatants from H1N1pdm-infected (C) or poly-I:C-treated (D) Usp25^{WT} and Usp25^{C178S} A549 cells (MOI=0.01) were harvested, and cytokine/chemokine profiles were determined by flow cytometry using the LEGENDplex antiviral and proinflammatory cytokine response panel. Data are shown as means of n = 3 ± standard deviations (SD). Data analyzed by Student's unpaired t test (*p < 0.1, **p < 0.01, ***p < 0.001, ****p < 0.0001).

See also Figure S5.

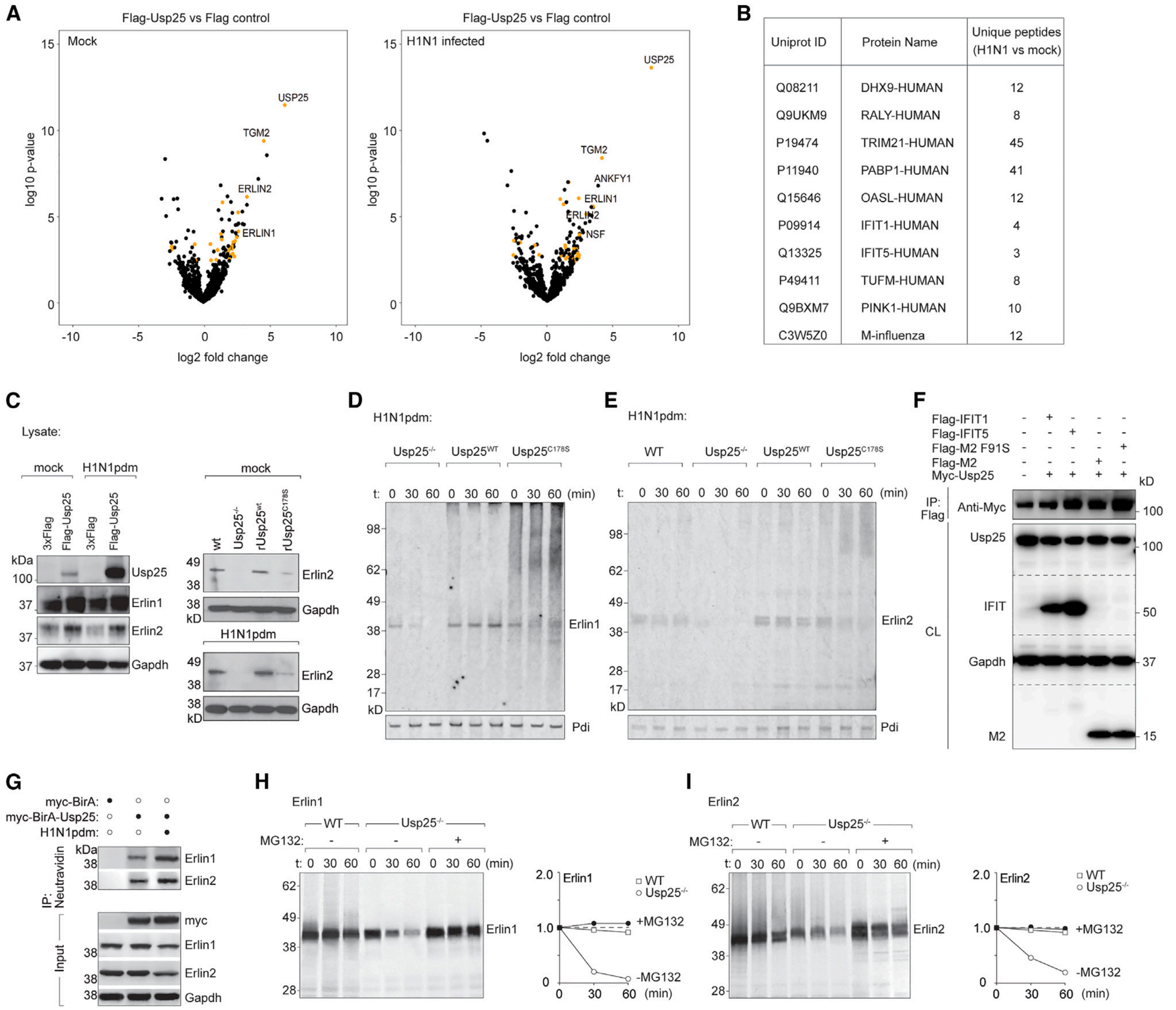

Uniprot ID	Protein Name	Unique peptides (H1N1 vs mock)
Q08211	DHX9-HUMAN	12
Q9UKM9	RALY-HUMAN	8
P19474	TRIM21-HUMAN	45
P11940	PABP1-HUMAN	41
Q15646	OASL-HUMAN	12
P09914	IFIT1-HUMAN	4
Q13325	IFIT5-HUMAN	3
P49411	TUFM-HUMAN	8
Q9BXM7	PINK1-HUMAN	10
C3W5Z0	M-influenza	12

Figure 6. Usp25 interacts with and stabilizes the Erlin1/2 complex

(A) FLAG-Usp25 expressed in A549 cells were either mock infected or H1N1pdm-infected (MOI = 1). Usp25 was immunoprecipitated from mock and H1N1pdm-infected cells on anti-FLAG M2 affinity beads and trypsin digested to identify interactors using mass spectrometry. Volcano plots indicate protein enrichments in mock and H1N1pdm-infected cells.

(B) Candidates with the highest number of unique peptides were isolated specifically from H1N1pdm-infected cells.

(C) A549 cells expressing either FLAG-tag or FLAG-Usp25^{WT} were either mock infected or H1N1pdm infected. Expression of Erlin1 and Erlin2 were measured by immunoblotting. Gapdh levels measured as loading control. A549 cells either Usp25$^{-/-}$, or expressing Usp25^{WT} or Usp25^{C178S} were mock or H1N1pdm infected, and Erlin2 expression measured by immunoblotting.

(D and E) IAV-infected A549 cells either Usp25$^{-/-}$ or expressing Usp25^{WT} or Usp25^{C178S} were pulsed with [^{35}S]cysteine/methionine and chased for indicated time intervals. At each time point, Erlin1/2 was immunoprecipitated, resolved by SDS-PAGE and detected by autoradiography.

(F) HEK-293T cell extracts, transiently co-transfected with Myc-Usp25 together with the indicated plasmids encoding FLAG-IFIT1, FLAG-IFIT5, influenza FLAG-M2, and FLAG-M2 F91S (containing a mutation in its LC3-interacting region to prevent association with autophagosomes) were immunoprecipitated on anti-FLAG M2 affinity beads. The immunoprecipitates were analyzed by immunoblotting with anti-Myc to validate their interaction with Usp25. Transfected FLAG-tagged proteins were detected by immunoblotting the cell lysates (CL) with anti-FLAG. Gapdh was used as loading control.

(G) A549 cells expressing either myc-BirA or myc-BirA-Usp25 cultured in biotin-supplemented media were either mock or H1N1pdm-infected. Lysates were captured on Neutravidin beads and eluates were immunoblotted for Erlin1 and Erlin2.

(H and I) IAV-infected WT and Usp25$^{-/-}$ cells were pulsed with [^{35}S]cysteine/methionine for 10 min and chased in cold media for indicated time intervals in DMSO alone or 50 μM MG132-treated (o/n) cells. Erlin1 and Erlin2 were immunoprecipitated, resolved by SDS-PAGE and detected by autoradiography. Quantitation was performed as relative amounts normalized to the starting amount at t = 0.

See also Figure S6.

of Usp25 followed by capture on Neutravidin beads and immunoblotting for Erlin1/2 (Figure 6G). To verify if Usp25-dependent turnover of Erlin1/2 occurred via proteasomal degradation, we performed pulse-chase analyses in [^{35}S]cysteine/methionine-labeled infected wild-type and Usp25$^{-/-}$ cells, combined with MG132 treatment to block proteasomal degradation. Erlin1/2 turnover in Usp25$^{-/-}$ cells could be rescued upon MG132 treatment, verifying proteasomal degradation of this complex (Figures 6H and 6I). Although mitochondrial components (e.g., Tufm) were identified as Usp25 interactors, their expression levels were not appreciably altered in wild-type and Usp25$^{-/-}$ cells or in Usp25^{WT} and Usp25^{C178S} during the course of infection with IAV (Figures S6A–S6C). In addition, a very modest increase in mitochondrial localization was observed for Usp25 in infected cells (Figures S6D and S6E). Collectively, our data indicated that, although Usp25 interacted with mitochondrial components in virus-infected cells, it had a more direct impact on the stability of the Erlin1/2 complex.

Usp25-Erlin1/2 serves to restrict virus production by regulating cholesterol flux

To determine how Usp25-Erlin1/2 restricted influenza infection, we first generated shRNA-mediated depletions of Erlin1, Erlin2, and a combination of Erlin1/2 (Figure 7A). Control and Erlin1/2-depleted cells were infected with IAV. In line with the Usp25-deficient phenotype, Erlin1/2-deficient cells displayed increased production of virus particles (Figure 7B). This phenomenon was recapitulated in cells infected with a wide range of viruses, including the highly pathogenic H5N1 and SARS-CoV-2 (Figure 7C). In contrast, overexpression of wild-type Erlin1/2 resulted in significantly reduced virus production, whereas a spastic paraplegia-linked mutant variant (Erlin^{T65I}) did not affect virus production, confirming its role as a virus restriction complex (Figure 7D). The activity of Usp25-dependent virus restriction via Erlin1/2 was also verified by overexpression of Usp25 in the Erlin1/2-depleted cells, which failed to restrict H1N1pdm infection (Figure 7E). On the other hand, overexpression of Erlin2 in Usp25-deficient cells resulted in restriction of H1N1pdm infection (Figures S7A and S7B).

A previous study implicated Erlin1/2 as interactors of Srebp2,[34] a key transcription factor that regulates cholesterol biosynthesis. Therefore, to determine whether Usp25-deficient and Erlin1/2-deficient cells displayed altered lipid and cholesterol content, we first stained for lipid droplets in them. No significant difference was observed in the abundance of lipid droplets in Usp25-deficient or Erlin1/2-depleted cells (Figure 7F). However, we detected significantly increased activation of Srebp2 in infected Usp25$^{-/-}$ and Usp25^{C178S} mutant cells, but not in Usp25^{WT} cells. Similarly, Erlin1/2-depleted cells displayed increased SREBP activation, measured by metabolic labeling with [^{35}S]cysteine/methionine (Figures 7G and 7H). In line with these observations, we measured HMG-CoA reductase levels (the rate-limiting enzyme in cholesterol biosynthesis) in infected Usp25-deficient and Erlin1/2-depleted cells, both of which displayed increased levels compared with controls (Figure 7I). This was also supported by increased total cholesterol levels in these cells compared with controls (Figure 7I). Furthermore, we detected significantly increased [^{3}H]cholesterol efflux in the Usp25-deficient and Erlin1/2-deficient cells (Figure 7J). Depletion of intracellular cholesterol levels by treatment with either methyl β-cyclodextrin or by simvastatin resulted in impaired virus production as well as surface levels of viral HA (Figures S7C–S7E). In line with the Usp25-deficient phenotype, loss of the Erlin1/2 complex also resulted in increased abundance of Rab11 and LC3 compartments, with a concomitant decrease in lysosomal proteins (Figure S7F), which could be reversed by inhibiting cholesterol synthesis (Figure S7G). Similarly, Erlin1/2 depletion facilitated virus-triggered autophagosome formation (with a decrease in autolysosome abundance), which was suppressed by inhibiting cholesterol synthesis (Figure S7H). Collectively, our results indicate that in infected cells, Usp25-dependent stabilization of the Erlin1/2 complex limits cholesterol biosynthetic flux and subsequent efflux, which potentially results in suppression of virus production. In an analogous model, limiting cholesterol synthesis was shown to trigger IFN-I signaling in an atherosclerosis model.[35] Our data indicate that Usp25-deficiency results in rapid turnover of the Erlin1/2 complex, resulting in unrestricted cholesterol and lipid biosynthesis to generate transport vesicles (e.g., Rab11 and LC3) that are hijacked by viruses for assembly and secretion from infected cells, and suppressing the production of TLR3-dependent inflammatory cytokines (Figure 7K).

DISCUSSION

In this study, we have identified a key role of Usp25 in limiting cholesterol biosynthetic flux to trigger TLR3-dependent immune activation and restrict virus infection. We previously identified Usp25 as a DUB that was activated upon virus infection[9]; here, we characterize its role in triggering a metabolic inflammatory circuit that serves as an important component of host defense. Viruses have been shown to manipulate intracellular lipids as resources for replication, assembly, and secretion.[19,25,36,37] Decreasing lipid synthesis during infection therefore serves to limit the resources available to viruses. The mevalonate pathway and IFN-I signaling pathway were proposed to be part of a metabolic inflammatory circuit[35]; our data indicate that this circuit is regulated by the Usp25-Erlin1/2 complex in order to maintain appropriate immune responses to infection.

IAV is known to induce autophagy by promoting autophagosome generation while inhibiting fusion to form autolysosomes, resulting in an accumulation of LC3-II to facilitate viral proliferation.[27] Viral M2, HA, and NS1 proteins are able to induce autophagy. Furthermore, the M2 protein was reported to subvert autophagy to generate LC3+ compartments for transport to viral budding sites at the plasma membrane.[21] Viral manipulation of this pathway therefore serves multiple purposes. Our data indicate that loss of the Usp25-Erlin1/2 dependent restriction results in increased formation of LC3+ compartments, thereby creating an intracellular environment conducive to infection. In a similar vein, Rab11 compartments have been shown to facilitate transport of viral RNP to viral assembly and budding sites at the plasma membrane.[22] We show that increased cholesterol biosynthesis alone is sufficient to trigger the formation of Rab11 compartments, again providing a permissive environment to virus assembly. This increases virus production in Usp25$^{-/-}$ cells by contributing to the later stages of the viral

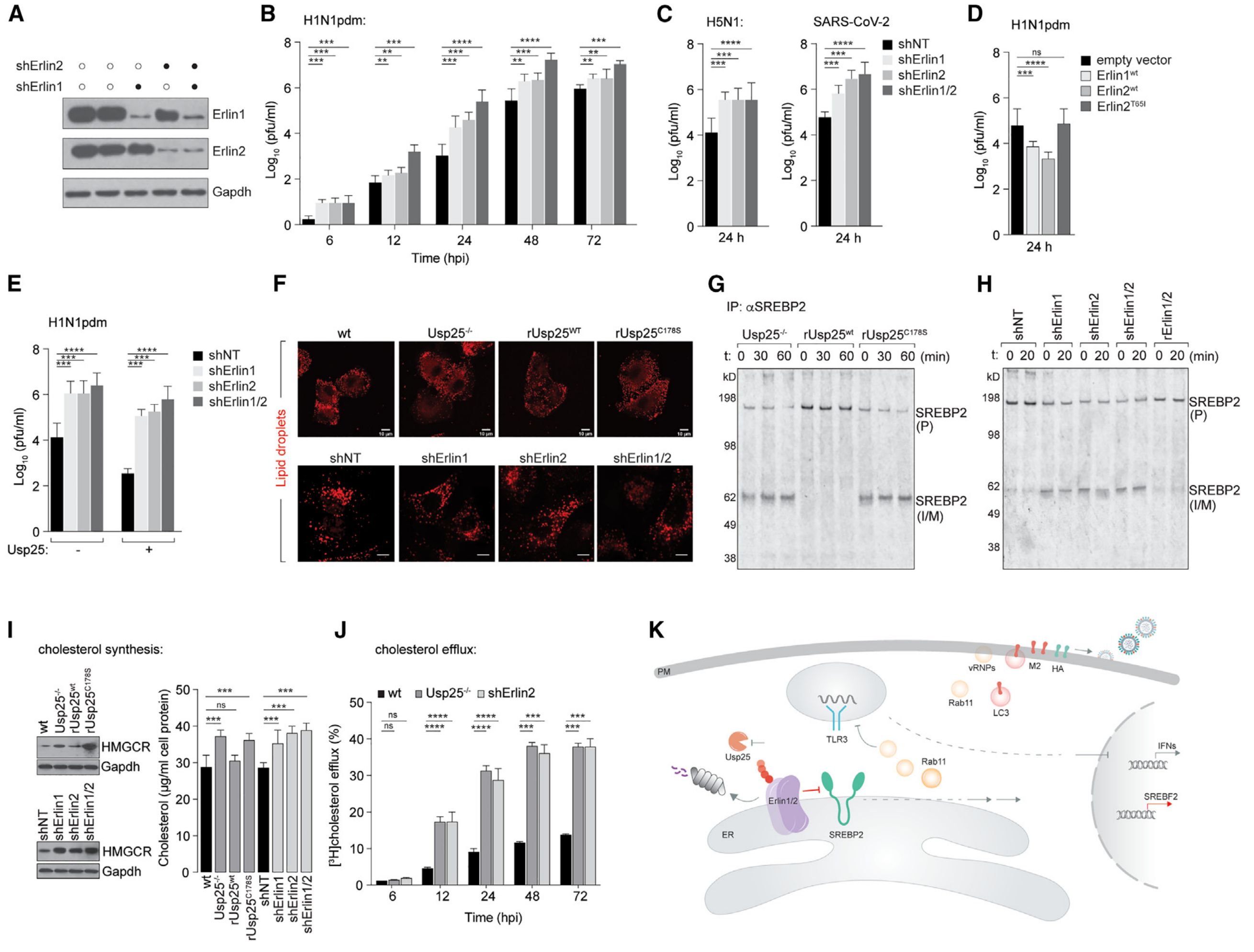

Figure 7. Usp25 stabilizes the Erlin1/2 complex to regulate cholesterol biosynthetic flux

(A) shRNA-mediated gene-depletion was performed for Erlin1 or Erlin2 or both in A549 cells and verified by immunoblotting.

(B and C) Erlin1/2-depleted cells were challenged with H1N1pdm, H5N1, and SARS-CoV-2 for indicated time intervals. Viral titers were measured using plaque assays.

(D) A549 cells expressing empty vector control, $Erlin1^{WT}$, $Erlin2^{WT}$, and $Erlin2^{T65I}$ were challenged with H1N1pdm (MOI = 0.01) and viral titers measured by plaque assay 24 h post infection.

(E) Usp25 over-expressed in Erlin1/2-depleted cells from (A) were infected with H1N1pdm (MOI 0.01) for 48 h. Viral titers were measured using plaque assay.

(F) WT, $Usp25^{-/-}$, $Usp25^{WT}$, or $Usp25^{C178S}$ A549 cells were stained for lipid droplets using BODIPY labeling upon mock and H1N1pdm infection (upper panel). Erlin1/2-depleted cells were stained for lipid droplets using BODIPY labeling upon mock and H1N1pdm infection (lower panel).

(G) IAV-infected $Usp25^{-/-}$, $Usp25^{WT}$, or $Usp25^{C178S}$ A549 cells were pulsed with [^{35}S]cysteine/methionine and chased for indicated time intervals. SREBP2 was immunoprecipitated at each time point, resolved by gel electrophoresis and detected by autoradiography.

(H) Same as (G) in Erlin1/2-depleted cells.

(I) Lysates generated from infected WT, $Usp25^{-/-}$, $Usp25^{WT}$, or $Usp25^{C178S}$ A549 cells (upper panel) and from Erlin1, Erlin2, and Erlin1/2 depleted cells (lower panel) were immunoblotted for HMG-CoA reductase (HMGCR) levels. Total cellular cholesterol levels in indicated cells were measured by lipid extraction into hexane/isopropanol (vol/vol, 3:2) followed by Amplex Red cholesterol quantitation as per the manufacturer's protocol.

(J) Cholesterol efflux in infected cells was measured in supernatants collected from [^{3}H]cholesterol labeled WT, $Usp25^{-/-}$ and shErlin2 cells by scintillation counting.

(K) Model for Usp25-mediated stabilization of the Erlin1/2 complex and regulation of cholesterol biosynthetic flux.

See also Figure S7.

life cycle of assembly and secretion. Collectively, altered lipid biosynthesis creates an ideal environment for viral propagation.

A key implication of our data is that viral sensing can occur by altering intracellular lipid content that is directly conveyed to inhibit *de novo* lipid synthesis and engage innate sensors to produce IFN-I. This most likely occurs due to altered cholesterol pools in intracellular compartments that are sensed by innate immune receptors. Although influenza viruses are largely recognized by RIG-I and TLR3, our data indicate that altered cholesterol pools are sensed via TLR3 and not by RIG-I. Conceptually, this makes sense, given the location of TLR3 in endosomal membranes as opposed to RIG-I in the cytoplasm. These

findings are in line with our own previous studies showing decreased synthesis and increased esterification of lipids upon exogenous IFN-stimulation.[38]

Usp25 was previously implicated in innate immune signaling in mouse models, where Usp25 deficiency resulted in increased susceptibility to infection.[11] Our results are in line with these findings and provide the mechanistic underpinning of Usp25-dependent innate immune activation. More importantly, limiting cholesterol and fatty acid synthesis is expected to be a universal feature in inflammatory responses to infection as restriction mechanisms. Indeed, Erlin1 emerged as a host restriction factor for SARS-CoV-2.[39] Targeting molecules such as the Smurf1 E3-ligase,[40,41] that functions as a negative regulator of Usp25[42] to boost host defense, will therefore be a promising approach to developing potential antivirals.

Limitations of the study

The mechanism underpinning increased production of a subset of cytokines from the Usp25$^{-/-}$ cells is currently unknown, and the physiological significance of the differences in responses of classical inflammatory cytokines versus this subset is not entirely clear.

It was not possible to visualize the precise localization of Usp25 with that of Erlin1/2 in virus-infected cells. Although our biochemical data indicate an increase in association in infected cells compared with mock infection, visualization would have enabled analyses of Usp25 distribution in infected cells and the bystander population.

In this study, virus infections were performed in 2D cultures of cell lines, which differ from the tissue architecture in the lung.

STAR★METHODS

Detailed methods are provided in the online version of this paper and include the following:

- KEY RESOURCES TABLE
- RESOURCE AVAILABILITY
  - Lead Contact
  - Materials Availability
  - Data and Code Availability
- EXPERIMENTAL MODEL AND STUDY PARTICIPANT DETAILS
  - Cell lines
  - Virus stocks
- METHOD DETAILS
  - Virus infection
  - Real-time reverse transcription quantitative PCR (RT-qPCR)
  - Plaque assays
  - Poly (I:C) stimulation
  - Deubiquitylase activity
  - Generation of knock-out and knock-down cells
- QUANTIFICATION AND STATISTICAL ANALYSIS

SUPPLEMENTAL INFORMATION

Supplemental information can be found online at https://doi.org/10.1016/j.devcel.2023.08.013.

ACKNOWLEDGMENTS

This work was supported by research grants from the Research Grants Council of the Hong Kong Special Administrative Region (17113019) (S.S.), Health and Medical Research Funds (19180912) (S.S.), the Wellcome Trust (220776/Z/20/Z and 223107/Z/21/Z) (S.S.), and partially by the InnoHK funding (C2i) administered by the Innovation and Technology Commission (S.S. and Y.L.).

AUTHOR CONTRIBUTIONS

Q.W.T., H.H.W., T.H., V.S., J.H., A.H., H.L., Y.L., C.K.P.M., L.Y.L.S., and R.U. performed and analyzed experiments. S.S. designed and conducted the study and wrote the manuscript.

DECLARATION OF INTERESTS

The authors declare no competing interests.

INCLUSION AND DIVERSITY

We support the inclusive, diverse, and equitable conduct of research.

Received: June 14, 2022
Revised: May 20, 2023
Accepted: August 9, 2023
Published: September 7, 2023

REFERENCES

1. Iuliano, A.D., Roguski, K.M., Chang, H.H., Muscatello, D.J., Palekar, R., Tempia, S., Cohen, C., Gran, J.M., Schanzer, D., Cowling, B.J., et al. (2018). Estimates of global seasonal influenza-associated respiratory mortality: a modelling study. Lancet *391*, 1285–1300. https://doi.org/10.1016/S0140-6736(17)33293-2.
2. Meganck, R.M., and Baric, R.S. (2021). Developing therapeutic approaches for twenty-first-century emerging infectious viral diseases. Nat. Med. *27*, 401–410. https://doi.org/10.1038/s41591-021-01282-0.
3. Jiang, X., and Chen, Z.J. (2011). The role of ubiquitylation in immune defence and pathogen evasion. Nat. Rev. Immunol. *12*, 35–48. https://doi.org/10.1038/nri3111.
4. Munnur, D., Teo, Q., Eggermont, D., Lee, H.H.Y., Thery, F., Ho, J., van Leur, S.W., Ng, W.W.S., Siu, L.Y.L., Beling, A., et al. (2021). Altered ISGylation drives aberrant macrophage-dependent immune responses during SARS-CoV-2 infection. Nat. Immunol. *22*, 1416–1427. https://doi.org/10.1038/s41590-021-01035-8.
5. Shin, D., Mukherjee, R., Grewe, D., Bojkova, D., Baek, K., Bhattacharya, A., Schulz, L., Widera, M., Mehdipour, A.R., Tascher, G., et al. (2020). Papain-like protease regulates SARS-CoV-2 viral spread and innate immunity. Nature *587*, 657–662. https://doi.org/10.1038/s41586-020-2601-5.
6. Karim, M., Biquand, E., Declercq, M., Jacob, Y., van der Werf, S., and Demeret, C. (2020). Nonproteolytic K29-linked ubiquitination of the PB2 replication protein of influenza A viruses by proviral cullin 4-based E3 ligases. mBio *11*, e00305-20. https://doi.org/10.1128/mBio.00305-20.
7. Liao, T.L., Wu, C.Y., Su, W.C., Jeng, K.S., and Lai, M.M.C. (2010). Ubiquitination and deubiquitination of NP protein regulates influenza A virus RNA replication. EMBO J. *29*, 3879–3890. https://doi.org/10.1038/emboj.2010.250.
8. Jahan, A.S., Lestra, M., Swee, L.K., Fan, Y., Lamers, M.M., Tafesse, F.G., Theile, C.S., Spooner, E., Bruzzone, R., Ploegh, H.L., et al. (2016). Usp12 stabilizes the T-cell receptor complex at the cell surface during signaling. Proc. Natl. Acad. Sci. USA *113*, E705–E714. https://doi.org/10.1073/pnas.1521763113.
9. Jahan, A.S., Biquand, E., Muñoz-Moreno, R., Le Quang, A., Mok, C.K.-P., Wong, H.H., Teo, Q.W., Valkenburg, S.A., Chin, A.W.H., Man Poon, L.L., et al. (2020). OTUB1 is a key regulator of RIG-I-dependent immune signaling and is targeted for proteasomal degradation by influenza A

NS1. Cell Rep. *30*, 1570–1584.e6. https://doi.org/10.1016/j.celrep.2020.01.015.

10. Zhong, B., Liu, X., Wang, X., Chang, S.H., Liu, X., Wang, A., Reynolds, J.M., and Dong, C. (2012). Negative regulation of IL-17-mediated signaling and inflammation by the ubiquitin-specific protease USP25. Nat. Immunol. *13*, 1110–1117. https://doi.org/10.1038/ni.2427.
11. Lin, D., Zhang, M., Zhang, M.X., Ren, Y., Jin, J., Zhao, Q., Pan, Z., Wu, M., Shu, H.B., Dong, C., et al. (2015). Induction of USP25 by viral infection promotes innate antiviral responses by mediating the stabilization of TRAF3 and TRAF6. Proc. Natl. Acad. Sci. USA *112*, 11324–11329. https://doi.org/10.1073/pnas.1509968112.
12. Meulmeester, E., Kunze, M., Hsiao, H.H., Urlaub, H., and Melchior, F. (2008). Mechanism and consequences for paralog-specific SUMOylation of ubiquitin-specific protease 25. Mol. Cell *30*, 610–619. https://doi.org/10.1016/j.molcel.2008.03.021.
13. Sanyal, S., Ashour, J., Maruyama, T., Altenburg, A.F., Cragnolini, J.J., Bilate, A., Avalos, A.M., Kundrat, L., García-Sastre, A., and Ploegh, H.L. (2013). Type I interferon imposes a TSG101/ISG15 checkpoint at the Golgi for glycoprotein trafficking during influenza virus infection. Cell Host Microbe *14*, 510–521. https://doi.org/10.1016/j.chom.2013.10.011.
14. Kolpe, A., Arista-Romero, M., Schepens, B., Pujals, S., Saelens, X., and Albertazzi, L. (2019). Super-resolution microscopy reveals significant impact of M2e-specific monoclonal antibodies on influenza A virus filament formation at the host cell surface. Sci. Rep. *9*, 4450. https://doi.org/10.1038/s41598-019-41023-5.
15. Rossman, J.S., Jing, X., Leser, G.P., Balannik, V., Pinto, L.H., and Lamb, R.A. (2010). Influenza virus M2 ion channel protein is necessary for filamentous Virion formation. J. Virol. *84*, 5078–5088. https://doi.org/10.1128/JVI.00119-10.
16. Lan, Y., Van Leur, S.W., Fernando, J.A., Wong, H.H., Kampmann, M., Siu, L., Zhang, J., Li, M., Nicholls, J.M., and Sanyal, S. (2023). Viral subversion of selective autophagy is critical for biogenesis of virus replication organelles. Nat. Commun. *14*, 2698. https://doi.org/10.1038/s41467-023-38377-w.
17. Li, M.Y., Naik, T.S., Siu, L.Y.L., Acuto, O., Spooner, E., Wang, P., Yang, X., Lin, Y., Bruzzone, R., Ashour, J., et al. (2020). Lyn kinase regulates egress of flaviviruses in autophagosome-derived organelles. Nat. Commun. *11*, 5189. https://doi.org/10.1038/s41467-020-19028-w.
18. Zhang, J., Lan, Y., and Sanyal, S. (2017). Modulation of lipid droplet metabolism—A potential target for therapeutic intervention in Flaviviridae infections. Front. Microbiol. *8*, 2286. https://doi.org/10.3389/fmicb.2017.02286.
19. Wong, H.H., and Sanyal, S. (2020). Manipulation of autophagy by (+) RNA viruses. Semin. Cell Dev. Biol. *101*, 3–11. https://doi.org/10.1016/j.semcdb.2019.07.013.
20. Pombo, J.P., and Sanyal, S. (2018). Perturbation of intracellular cholesterol and fatty acid homeostasis during Flavivirus infections. Front. Immunol. *9*, 1276. https://doi.org/10.3389/fimmu.2018.01276.
21. Beale, R., Wise, H., Stuart, A., Ravenhill, B.J., Digard, P., and Randow, F. (2014). A LC3-interacting motif in the influenza A virus M2 protein is required to subvert autophagy and maintain Virion stability. Cell Host Microbe *15*, 239–247. https://doi.org/10.1016/j.chom.2014.01.006.
22. Alenquer, M., Vale-Costa, S., Etibor, T.A., Ferreira, F., Sousa, A.L., and Amorim, M.J. (2019). Influenza A virus ribonucleoproteins form liquid organelles at endoplasmic reticulum exit sites. Nat. Commun. *10*, 1629. https://doi.org/10.1038/s41467-019-09549-4.
23. Liu, X., Xu, F., Ren, L., Zhao, F., Huang, Y., Wei, L., Wang, Y., Wang, C., Fan, Z., Mei, S., et al. (2021). March8 inhibits influenza A virus infection by targeting viral M2 protein for ubiquitination-dependent degradation in lysosomes. Nat. Commun. *12*, 4427. https://doi.org/10.1038/s41467-021-24724-2.
24. Ao, X., Zou, L., and Wu, Y. (2014). Regulation of autophagy by the Rab GTPase network. Cell Death Differ. *21*, 348–358. https://doi.org/10.1038/cdd.2013.187.
25. Teo, Q.W., van Leur, S.W., and Sanyal, S. (2021). Escaping the Lion's Den: redirecting autophagy for unconventional release and spread of viruses. FEBS Journal *288*, 3913–3927. https://doi.org/10.1111/febs.15590.
26. Cawley, N.X., Sojka, C., Cougnoux, A., Lyons, A.T., Nicoli, E.R., Wassif, C.A., and Porter, F.D. (2020). Abnormal LAMP1 glycosylation may play a role in Niemann-Pick disease, type C pathology. PLoS One *15*, e0227829. https://doi.org/10.1371/journal.pone.0227829.
27. Li, J., Deffieu, M.S., Lee, P.L., Saha, P., and Pfeffer, S.R. (2015). Glycosylation inhibition reduces cholesterol accumulation in NPC1 protein-deficient cells. Proc. Natl. Acad. Sci. USA *112*, 14876–14881. https://doi.org/10.1073/pnas.1520490112.
28. Wu, W., Zhang, W., Duggan, E.S., Booth, J.L., Zou, M.H., and Metcalf, J.P. (2015). RIG-I and TLR3 are both required for maximum interferon induction by influenza virus in human lung alveolar epithelial cells. Virology *482*, 181–188. https://doi.org/10.1016/j.virol.2015.03.048.
29. Wrigley, J.D., Gavory, G., Simpson, I., Preston, M., Plant, H., Bradley, J., Goeppert, A.U., Rozycka, E., Davies, G., Walsh, J., et al. (2017). Identification and characterization of dual inhibitors of the USP25/28 deubiquitinating enzyme subfamily. ACS Chem. Biol. *12*, 3113–3125. https://doi.org/10.1021/acschembio.7b00334.
30. Gersch, M., Wagstaff, J.L., Toms, A.V., Graves, B., Freund, S.M.V., and Komander, D. (2019). Distinct USP25 and USP28 oligomerization states regulate deubiquitinating activity. Mol. Cell *74*, 436–451.e7. https://doi.org/10.1016/j.molcel.2019.02.030.
31. Sauer, F., Klemm, T., Kollampally, R.B., Tessmer, I., Nair, R.K., Popov, N., and Kisker, C. (2019). Differential oligomerization of the deubiquitinases USP25 and USP28 regulates their activities. Mol. Cell *74*, 421–435.e10. https://doi.org/10.1016/j.molcel.2019.02.029.
32. Lei, Y., Wen, H., Yu, Y., Taxman, D.J., Zhang, L., Widman, D.G., Swanson, K.V., Wen, K.W., Damania, B., Moore, C.B., et al. (2012). The mitochondrial proteins NLRX1 and TUFM form a complex that regulates Type I interferon and autophagy. Immunity *36*, 933–946. https://doi.org/10.1016/j.immuni.2012.03.025.
33. Sallam, T., Jones, M.C., Gilliland, T., Zhang, L., Wu, X., Eskin, A., Sandhu, J., Casero, D., Vallim, T.Q.de A., Hong, C., et al. (2016). Feedback modulation of cholesterol metabolism by the lipid-responsive non-coding RNA LeXis. Nature *534*, 124–128. https://doi.org/10.1038/nature17674.
34. Huber, M.D., Vesely, P.W., Datta, K., and Gerace, L. (2013). Erlins restrict SREBP activation in the ER and regulate cellular cholesterol homeostasis. J. Cell Biol. *203*, 427–436. https://doi.org/10.1083/jcb.201305076.
35. York, A.G., Williams, K.J., Argus, J.P., Zhou, Q.D., Brar, G., Vergnes, L., Gray, E.E., Zhen, A., Wu, N.C., Yamada, D.H., et al. (2015). Limiting cholesterol biosynthetic flux spontaneously engages Type I IFN signaling. Cell *163*, 1716–1729. https://doi.org/10.1016/j.cell.2015.11.045.
36. van Leur, S.W., Heunis, T., Munnur, D., and Sanyal, S. (2021). Pathogenesis and virulence of Flavivirus infections. Virulence *12*, 2814–2838. https://doi.org/10.1080/21505594.2021.1996059.
37. Zhang, J., Lan, Y., Li, M.Y., Lamers, M.M., Fusade-Boyer, M., Klemm, E., Thiele, C., Ashour, J., and Sanyal, S. (2018). Flaviviruses exploit the lipid droplet protein AUP1 to trigger lipophagy and drive virus production. Cell Host Microbe *23*, 819–831.e5. https://doi.org/10.1016/j.chom.2018.05.005.
38. Munnur, D., Banducci-Karp, A., and Sanyal, S. (2022). ISG15 driven cellular responses to virus infection. Biochem. Soc. Trans. *50*, 1837–1846. https://doi.org/10.1042/BST20220839.
39. Martin-Sancho, L., Lewinski, M.K., Pache, L., Stoneham, C.A., Yin, X., Becker, M.E., Pratt, D., Churas, C., Rosenthal, S.B., Liu, S., et al. (2021). Functional landscape of SARS-CoV-2 cellular restriction. Mol. Cell *81*, 2656–2668.e8. https://doi.org/10.1016/j.molcel.2021.04.008.
40. Franco, L.H., Nair, V.R., Scharn, C.R., Xavier, R.J., Torrealba, J.R., Shiloh, M.U., and Levine, B. (2017). The ubiquitin ligase Smurf1 functions in selective autophagy of Mycobacterium tuberculosis and anti-tuberculous Host Defense. Cell Host Microbe *21*, 59–72. https://doi.org/10.1016/j.chom.2016.11.002.

CellPress
OPEN ACCESS

41. Cao, Y., and Zhang, L. (2013). A Smurf1 tale: function and regulation of an ubiquitin ligase in multiple cellular networks. Cell. Mol. Life Sci. *70*, 2305–2317. https://doi.org/10.1007/s00018-012-1170-7.

42. Qian, G., Hu, X., Li, G., Ding, Y., Zhu, L., Zheng, H., Li, M., Li, Z., Pan, J., Li, Y., et al. (2018). Smurf1 restricts the antiviral function mediated by USP25 through promoting its ubiquitination and degradation. Biochem. Biophys. Res. Commun. *498*, 537–543. https://doi.org/10.1016/j.bbrc.2018.03.015.

43. Fan, Y., Mok, C.K.-P., Chan, M.C.W., Zhang, Y., Nal, B., Kien, F., Bruzzone, R., and Sanyal, S. (2017). Cell cycle-independent role of cyclin D3 in Host Restriction of Influenza Virus Infection. J. Biol. Chem. *292*, 5070–5088. https://doi.org/10.1074/jbc.M117.776112.

44. Chan, R.W.Y., Chan, L.L.Y., Mok, C.K.P., Lai, J., Tao, K.P., Obadan, A., Chan, M.C.W., Perez, D.R., Peiris, J.S.M., and Nicholls, J.M. (2017). Replication of H9 influenza viruses in the human ex vivo respiratory tract, and the influence of neuraminidase on virus release. Sci. Rep. *7*, 6208. https://doi.org/10.1038/s41598-017-05853-5.

45. Herfst, S., Schrauwen, E.J.A., Linster, M., Chutinimitkul, S., de Wit, E., Munster, V.J., Sorrell, E.M., Bestebroer, T.M., Burke, D.F., Smith, D.J., et al. (2012). Airborne transmission of influenza A/H5N1 virus between ferrets. Science *336*, 1534–1541. https://doi.org/10.1126/science.1213362.

46. Mok, C.K.P., Lee, H.H.Y., Lestra, M., Nicholls, J.M., Chan, M.C.W., Sia, S.F., Zhu, H., Poon, L.L.M., Guan, Y., and Peiris, J.S.M. (2014). Amino acid substitutions in polymerase basic Protein 2 gene contribute to the pathogenicity of the novel A/H7N9 influenza virus in mammalian hosts. J. Virol. *88*, 3568–3576. https://doi.org/10.1128/JVI.02740-13.

47. Lv, Z., Deng, Y.Q., Ye, Q., Cao, L., Sun, C.Y., Fan, C., Huang, W., Sun, S., Sun, Y., Zhu, L., et al. (2020). Structural basis for neutralization of SARS-CoV-2 and SARS-CoV by a potent therapeutic antibody. Science *369*, 1505–1509. https://doi.org/10.1126/science.abc5881.

48. Lo, H.S., Hui, K.P.Y., Lai, H.M., He, X., Khan, K.S., Kaur, S., Huang, J., Li, Z., Chan, A.K.N., Cheung, H.H.-Y., et al. (2021). Simeprevir potently suppresses SARS-CoV-2 replication and synergizes with remdesivir. ACS Cent. Sci. *7*, 792–802. https://doi.org/10.1021/acscentsci.0c01186.

49. Xu, D., Liu, J., Fu, T., Shan, B., Qian, L., Pan, L., and Yuan, J. (2017). USP25 regulates Wnt signaling by controlling the stability of tankyrases. Genes Dev. *31*, 1024–1035. https://doi.org/10.1101/gad.300889.117.

50. te Velthuis, A.J.W., Long, J.C., Bauer, D.L.V., Fan, R.L.Y., Yen, H.L., Sharps, J., Siegers, J.Y., Killip, M.J., French, H., Oliva-Martín, M.J., et al. (2018). Mini viral RNAs act as innate immune agonists during influenza virus infection. Nat. Microbiol. *3*, 1234–1242. https://doi.org/10.1038/s41564-018-0240-5.

51. Sanyal, S., Claessen, J.H.L., and Ploegh, H.L. (2012). A viral deubiquitylating enzyme restores dislocation of substrates from the endoplasmic reticulum (ER) in semi-intact cells. J. Biol. Chem. *287*, 23594–23603. https://doi.org/10.1074/jbc.M112.365312.

52. Doench, J.G., Fusi, N., Sullender, M., Hegde, M., Vaimberg, E.W., Donovan, K.F., Smith, I., Tothova, Z., Wilen, C., Orchard, R., et al. (2016). Optimized sgRNA design to maximize activity and minimize off-target effects of CRISPR-Cas9. Nat. Biotechnol. *34*, 184–191. https://doi.org/10.1038/nbt.3437.

STAR★METHODS

KEY RESOURCES TABLE

REAGENT or RESOURCE	SOURCE	IDENTIFIER
Antibodies		
Anti-Usp25	Abcam	Cat# ab187156
Anti-SMURF1	Santa Cruz	Cat# sc-100616
Anti-Usp28	Abcam	Cat# ab126604; RRID: AB_11127442
Anti-GAPDH	Abcam	Cat# ab8245; RRID: AB_2107448
Anti-PB2	Thermo Fisher Scientific	Cat# PA5-32220; RRID: AB_2549693
Anti-HA	Abcam	Cat# ab119966; RRID: AB_10899358
Anti-NS1	Santa Cruz	Cat# sc-130568; RRID: AB_2011757
Anti-NP	Abcam	Cat# ab128193; RRID: AB_11143769
Anti-RIG-I	Abcam	Cat# ab180675
Anti-MAVS	Cell Signaling Technology	Cat# 3993; RRID: AB_823565
Anti-TBK1	Cell Signaling Technology	Cat# 3504; RRID: AB_2255663
Anti-Phospho-TBK1 (Ser172)	Cell Signaling Technology	Cat# 5483; RRID: AB_10693472
TRAF3	Abcam	Cat# 36988
Anti-IRF3	Cell Signaling Technology	Cat# 4302; RRID: AB_1904036
Anti-Phospho-IRF3 (Ser386)	Cell Signaling Technology	Cat# 37829; RRID: AB_2799121
Anti-IRF7	Cell Signaling Technology	Cat# 4920; RRID: AB_2127551
Anti-STAT1	Cell Signaling Technology	Cat# 9172; RRID: AB_2198300
Anti-Phospho-STAT1 (Tyr701)	Cell Signaling Technology	Cat# 9167; RRID: AB_561284
Anti-STAT2	Cell Signaling Technology	Cat# 4594; RRID: AB_2271323
IFIT1	Abcam	Cat# 236256
Anti-Phospho-STAT2 (Tyr690)	Cell Signaling Technology	Cat# 88410; RRID: AB_2800123
Anti-Beclin 1	Cell Signaling Technology	Cat# 3495; RRID: AB_1903911
Anti-ATG 5	Cell Signaling Technology	Cat 12994; RRID: AB_2630393
Anti-ATG 12	Cell Signaling Technology	Cat# 4180; RRID: AB_1903898
Anti-LC 3	Cell Signaling Technology	Cat# 2775; RRID: AB_915950
Anti-LAMP1	Cell Signaling Technology	Cat# 9091; RRID: AB_2687579
Anti-LAMP2	Abcam	Cat# 25631
Anti-Capthesin B	Cell Signaling Technology	Cat#31718; RRID: AB_2687580
Anti-Capthesin D	Abcam	Cat# ab198326
Anti-Rab 11	Cell Signaling Technology	Cat# 5589; RRID: AB_10693925
Anti-Rab 8b	Thermo Fisher Scientific	CAT# PA5-67354; RRID: AB_2664303
Anti-Flag-HRP	Sigma Aldrich	Cat# A8592; RRID: AB_439702
Anti-HA-HRP	Abcam	Cat# ab128131; RRID: AB_11143947
Anti-c-Myc	Roche	Cat# 11667203001; RRID: AB_390911
Bacterial and Virus Strains		
A/California/07/2009(H1N1 pandemic)	Jahan et al.[9]	N/A
A/Oklahoma/309/06 (H3N2)	Fan et al.[43]	N/A
A/Hong Kong/1073/99 (H9N2)	Chan et al.[44]	N/A
A/Vietnam/1203/2004 (H5N1)	Herfst et al.[45]	N/A
A/Shanghai/02/2013 (H7N9)	Mok et al.[46]	N/A
Human coronavirus 229E	ATCC	VR-740
BetaCoV/Hong Kong/VM20001061/2020	Lv et al.[47]	N/A
ZIKV (NC-14-5132)	Lan et al.[16] and Li et al.[17]	N/A
Chemocompetent E. coli DH5a	Thermo Fisher Scientific	Cat# 18265017

(Continued on next page)

Continued		
REAGENT or RESOURCE	SOURCE	IDENTIFIER
Chemicals, Peptides, and Recombinant Proteins		
DMEM	Thermo Fisher Scientific	Cat# 10569010
HEPES	Thermo Fisher Scientific	Cat# 15630130
Trypsin-EDTA	Thermo Fisher Scientific	Cat# 25200072
Penicillin-Streptomycin	Thermo Fisher Scientific	Cat# 15140122
Fetal Bovine Serum (FBS)	Thermo Fisher Scientific	Cat# 10500064
Phosphate-buffered saline (PBS)	Thermo Fisher Scientific	Cat# 14040133
Puromycin	InvivoGen	Cat# ant-pr-1
Hygromycin B	Thermo Fisher Scientific	Cat# 10687010
Triton X-100	Sigma Aldrich	Cat# 93443
Complete protease inhibitor cocktail	Roche	Cat# 11836145001
Normal Goat Serum (NGS)	Thermo Fisher Scientific	Cat# 31873
MG132	Sigma Aldrich	Cat# M7449
TransITLT1 transfection reagent	Mirus	Cat# MIR2304
Lipofectamine 3000	Thermo Fisher Scientific	Cat# L3000008
Polyethylenimine (PEI)	Polysciences	Cat# 23966-1
DharmaFECT1	Dharmacon	Cat# T-2001-03
Octadecyl Rhodamine B Chloride (R18)	Thermo Fisher Scientific	Cat# O246
Fix Perm Kits	BD PharMingen	Cat# 554714
TPCK-Trypisn	Thermo Fisher Scientific	Cat# 20233
T4 ligase	New England Biolabs	Cat# M0202S
Endo H	New England Biolabs	Cat# P0702S
PNGase F	New England Biolabs	Cat# P0704S
Critical Commercial Assays		
TriFECTa DsiRNA Kit	Integrated DNA Technologies	Cat# hs.Ri.USP28.13
TriFECTa DsiRNA Kit	Integrated DNA Technologies	Cat# hs.Ri.SMURF1.13
TriFECTa DsiRNA Kit	Integrated DNA Technologies	Cat# hs.Ri.BECN1.13
TriFECTa DsiRNA Kit	Integrated DNA Technologies	Cat# hs.Ri.MAP1LC3B.13
PrimeScript RT Reagent Kit	TAKARA	Cat# RR037A
MiniBEST Universal RNA Extraction Kit	TAKARA	Cat# 9767
MiniBEST Viral RNA Extraction Kit	TAKARA	Cat# 9766
TB Green Premix Ex Taq II (Tli RNase H Plus)	TAKARA	Cat# RR820A
LEGENDplex Human Anti-Virus Response Panel	Biolegend	Cat# 740390
LEGENDplex Human Proinflammatory Chemokine Panel 1	Biolegend	Cat# 740984
Deposited Data		
Proteomics; PRIDE database	This study	Accession: PXD034797
Experimental Models: Cell Lines		
A549	ATCC	CCL-185
A549 Usp25$^{-/-}$	This study	N/A
A549 Usp25^{WT}	This study	N/A
A549 Usp25^{C178S}	This study	N/A
Tandem-tagged mCherry-GFP-LC3 A549	This study	N/A
Tandem-tagged mCherry-EGFP-LC 3 A549 Usp25$^{-/-}$	This study	N/A
293T	ATCC	CRL-3216
A549 ACE2	Lo et al.[48]	N/A
MDCK	ATCC	CCL-34

(*Continued on next page*)

Continued

REAGENT or RESOURCE	SOURCE	IDENTIFIER
MRC-5	ATCC	CCL-171
Vero	ATCC	CCL-81
Oligonucleotides		
GGCACCAAGGCACATAACGG (sgRNA)	This study	N/A
GAGACTGAAAGATTACCTCA (sgRNA)	This study	N/A
AGCAAAAGCAGG (uni-12)	This study	N/A
AGTAGAAACAAGG (uni-13)	This study	N/A
GACCAATCCTGTCACCTCTGA (M Gene-Forward)	This study	N/A
AGGGCATTTTGGACAAAGCGTCTA (M Gene-Reverse)	This study	N/A
CACCATTGGCAATGAGCGGTTC (β-actin-Forward)	This study	N/A
AGGTCTTTGCGGATGTCCACGT (β-actin-Reverse)	This study	N/A
Recombinant DNA		
pSpCas9(BB)-2A-Puro (PX459) V2.0	Addgene	Addgene plasmid #62988
Usp25-PX459	This study	N/A
Flag-tagged-Usp25	Xu et al.[49]	N/A
Flag-tagged-Usp25 (C178S)	Xu et al.[49]	N/A
Myc-tagged- RIG I	te Velthuis et al.[50]	N/A
Flag-tagged-M2	This study	N/A
3xFlag IFIT1	Addgene	Addgene plasmid #53554
3xFlag IFIT5	Addgene	Addgene plasmid #53556
HA-Ubiquitin	Addgene	Addgene plasmid #18712
HA-Ubiquitin-K6	Addgene	Addgene plasmid #22900
HA-Ubiquitin-K11	Addgene	Addgene plasmid #22901
HA-Ubiquitin-K27	Addgene	Addgene plasmid #22902
HA-Ubiquitin-K29	Addgene	Addgene plasmid #22903
HA-Ubiquitin-K33	Addgene	Addgene plasmid #17607
HA-Ubiquitin-K48	Addgene	Addgene plasmid #17605
HA-Ubiquitin-K63	Addgene	Addgene plasmid #17606
mCherry-GFP-LC3	Addgene	Addgene plasmid #110060
Myc-tagged-SMURF1	Addgene	Addgene plasmid #13676
Flag-tagged-SMURF1 (C699A)	Addgene	Addgene plasmid #11753
VSV-G	Addgene	Addgene plasmid #8454
Software and Algorithms		
Prism 8.0	GraphPad Software	https://www.graphpad.com/scientific-software/prism/
ImageJ	NIH	https://imagej.net/ImageJ
ZEN confocal software	Zeiss	https://www.zeiss.com/microscopy/int/products/microscope-software/zen.html
FlowJo	Flowjo	https://www.flowjo.com/solutions/flowjo/downloads
Legendplex v8.0	Biolegend	https://www.biolegend.com/en-us/legendplex
Ingenuity Pathway Analysis software	QIAGEN	https://digitalinsights.qiagen.com/products-overview/discovery-insights-portfolio/analysis-and-visualization/qiagen-ipa/

CellPress
OPEN ACCESS

RESOURCE AVAILABILITY

Lead Contact

Further information and requests for resources and reagents should be directed to and will be fulfilled by the Lead Contact, Sumana Sanyal (sumana.sanyal@path.ox.ac.uk).

Materials Availability

All unique/ stable reagents generated in this study are available from the Lead Contact with a completed Materials Transfer Agreement.

Data and Code Availability

- The proteome dataset has been deposited in the PRIDE database and is publicly available as of the date of publication. Accession numbers are listed in the Key resources table. Original western blot images and microscopy data reported in this paper will be shared by the lead contact upon request.
- This paper does not report original code.
- Any additional information required to reanalyse the data reported in this paper is available from the lead contact upon request.

EXPERIMENTAL MODEL AND STUDY PARTICIPANT DETAILS

Cell lines

The following cell lines were used in this study: A549 (human; sex: male; ATCC-CCL-185), HEK293T (human; sex: unspecified), MDCK (*Canis familiaris*; sex: unspecified; ATCC-CCL-34) and Vero cells (*Cercopithecus aethiops*; sex: unspecified; ATCC-CCL-81) were obtained from commercial sources noted in the Key Resources Table and gender of the cell line was not a consideration in the study. Cells were maintained in Dulbecco's Modified Eagle Medium (DMEM; GIBCO) supplemented with 10% Fetal Bovine Serum (FBS; Thermo Fisher Scientific) and 1% Penicillin/Streptomycin (P/S; Thermo Fisher Scientific) at 37°C in a humidified incubator under 5% CO_2 supply.

Virus stocks

The following virus strains were used in this study: A/California/07/2009 (H1N1 pandemic), A/Oklahoma/309/06 (H3N2), A/Hong Kong/1073/99 (H9N2), H5N1 and Zika virus (NC-14-5132). Influenza viruses were propagated in Madin Darby Canine Kidney (MDCK) cells in DMEM supplemented with 1 μg/ml Tosyl Phenylalanyl Chloromethyl Ketone (TPCK)-treated trypsin (Thermo Scientific). Supernatants from the infected MDCK cells were harvested three days post-infection. In order to maintain the homogeneity of the viruses, they were propagated with limited passage number and seed stocks of viruses were prepared for future propagation. Virus stocks were aliquoted and stored at -80°C, and the viral titers were measured using plaque assay. Zika virus was titrated by determining the tissue culture infective dose 50% ($TCID_{50}$) in Vero cells infected with 10-fold serial dilutions of infectious supernatants for 90 min at 37°C.

METHOD DETAILS

Virus infection

Cells were seeded in 12-well plates with the seeding density of 0.2 x 10^6 cells/well one day in advance. Cells were infected with viruses at a multiplicity of infection (MOI) of 5 for single-cycle infection assay or 0.01 for multi-cycle infection assay. After an hour of adsorption, the viral inoculum was removed, and the infected cells were washed with Phosphate-buffered Saline (PBS; Thermo Fisher Scientific) and maintained in DMEM supplemented with 0.2 μg/ml TPCK-treated trypsin at 37°C. Supernatants, cell lysates, and total RNA were harvested at indicated post infection time points, cleared by centrifugation and stored at -80°C until further analysis.

Real-time reverse transcription quantitative PCR (RT-qPCR)

Total RNA was extracted from cells using MiniBEST Universal RNA Extraction Kit (Takara) according to the manufacturer's manual. One microgram of total RNA was used for the following reverse transcription assays. For the quantification of vRNA/ cRNA, 10 μM of uni-12/ uni-13 primer and 10 μM of β-actin specific primer complementary to the 3' β-actin gene were used together with PrimeScript RT Reagent Kit (Takara); while for mRNA measurement oligo-dT primer was used in the reverse transcription reaction. SYBR Green based real time (Roche) was used to determine the M gene copy number of vRNA, cRNA, and mRNA, and the number of β-actin mRNA was used to normalise the total RNA concentration between different samples. The PCR experiments were performed using LightCycler system 480 (Roche). A reaction mixture of 20 μl was composed of 1 μl of 10 μM forward primer, 1 μl of 10 μM reverse primer, 10 μl of SYBR Premix Ex Taq reagent (TAKARA), 5 μl of 10-fold diluted cDNA, and 3 μl of distilled water. The amplification program was as follows: 95°C for 5 minutes, followed by 45 cycles of PCR reaction of 95°C for 10 seconds, 60°C for 10 seconds, and 72°C for 10 seconds. The specificity of the assay was confirmed by melting curve analysis at the end of the amplification program. The primers for uni-12, uni-13, M gene and β-actin detection are described in the Key Resources Table.

Plaque assays

MDCK cells were seeded in 12-well plates with the seeding density of 0.5 x 10^6 cells/well one day in advance. The following day confluent MDCK cells were washed with PBS, and then incubated with 500 μl of the ten-fold serially diluted infectious media for an hour at 37°C. Afterwards, the viral inoculum was removed. Infected cells were washed twice with PBS, and overlaid with DMEM containing 1% agarose and 1 μg/ml TPCK-treated trypsin. The plates were incubated in inverted position at 37°C for three days. Cells were fixed with 4% formaldehyde in PBS overnight. Plaques were visualized by staining the plates with 1% crystal violet in 20% ethanol for 15 minutes. Plaques were counted and recorded after drying the plates.

Poly (I:C) stimulation

The endotoxin-free, high m.w. poly(I:C) was purchased from Invivogen (San Diego, CA). For RIG-I stimulation, 10 μg/ml poly(I:C) dissolved in 200 μl of serum-free medium was mixed with Lipofectamine and transfected according to the manufacturer's instructions, followed by incubation for 24 hours before analyzes. For TLR stimulation, 25μg/ml of poly(I:C) was mixed in complete medium, added to cells and incubated for 24 hours.

Deubiquitylase activity

~1 x 10^7 cells were detached from 10 cm dishes by brief trypsinization, washed once with Hank's balanced salt solution (HBSS) and resuspended in 100 μl (HBSS) on ice. PFO was added to cells to a final concentration of 100 nM and maintained on ice for 5 min.[8,51] The reaction mix was supplemented with an ATP regenerating mix, 10μM HA-Ubvme and protease inhibitor cocktail, and transferred to 37°C for 20 min. The reaction was terminated with lysis buffer. HA-Ubvme reactive DUBs were resolved by SDS-PAGE and detected by immunoblotting.

Generation of knock-out and knock-down cells

CRISPR/Cas9 Mediated Deletion of Usp25

Potential target sequence for CRISPR interference were found using the rules outlined elsewhere.[52] The sgRNAs were annealed and cloned into the chimeric CRISPR/Cas9 vector pX459 V2.0, which has a puromycin resistant cassette. Sequences of generated pX459-sgRNA clones were confirmed through Sanger sequencing. A549 cells were then transfected with the pX459/USP25 sgRNA plasmid and subjected to puromycin selection at a concentration of 4.0 μg/ml for 4 days. Single clones of putative knockout cells were then isolated by serial dilution, expanded, and validated for gene deletion using Western blot.

DsiRNA-Mediated Knockdown of Genes

DsiRNA against specific genes were purchased from Integrated DNA Technologies as TriFECTa DsiRNA kit. DsiRNA mixed with DharmaFECT1 reagent were added to cells in 12-well plates in DMEM supplemented with 10% FBS and incubated at 37°C for 24 hours.

Lentiviral transduction and reconstitution of knock-out cells

A549 cells expressing tandem-tagged mCherry-EGFP-LC3, flag-tagged wild type Usp25 ($Usp25^{WT}$) and flag-tagged Usp25 catalytic inactive mutant (C178S) ($Usp25^{C178S}$) were generated by lentiviral transduction. Plasmids of VSV-G, PLP1(GAG/POL), PLP2 and pLenti vector (plasmid of interest) were co-transfected in HEK293T cells for 24 hours. The supernatant was harvested and purified via passing a 0.22 μm syringe filter. The purified lentiviral suspension was added to wild type and $Usp25^{-/-}$ A549 cells for three days, followed by hygromycin selection at a concentration of 1.6 mg/ml for two weeks. Single clones of tandem-tagged mCherry-EGFP-LC3 stable cells, $Usp25^{WT}$ and $Usp25^{C178S}$ A549 cells were isolated, expanded and validated by Western blot.

Immunoprecipitation

Transient transfection was performed using PEI, and the DNA: PEI ratio was 1: 3. HEK293T cells that were grown in 6-well plates were co-transfected with 1 μg of Usp25 expression plasmid together with 1 μg of plasmids encoding the Myc-RIG-I, Flag-PB2 of H3N2, PR8, or WSN. Cell lysates were collected 24 hours post transfection. Immunoprecipitation was performed using anti-Flag M2 affinity gel (Sigma-Aldrich) according to manufacturers' protocols. The eluates were resuspended in 30 μl of 4× Laemmli Sample Buffer supplemented with β-mercaptoethanol and boiled at 95°C for 10 minutes. The immunoprecipitates were analyzed by Western blot. BirA*-Usp25 was transduced into A549 cells and either mock or H1N1pdm infected (MOI 0.01). Cells were grown in culture supplemented with 50 μM biotin. Lysates prepared from mock and infected cells were immunoprecipitated on Neutravidin beads, eluted into Laemmli buffer and immunoblotted with anti-Erlin1 and anti-Erlin2 antibodies.

In vivo *ubiquitylation assay*

Ubiquitylation assays were performed as previously described.[9] Briefly, HEK293T cells cultured in 60 mm dishes were co-transfected with 1 μg of HA-Ub, HA-K6-Ub, HA-K27-Ub, HA-K29-Ub, HA-K33-Ub, HA-K48-Ub, or HA-K63-Ub, Flag-M2 and Myc-Usp25 or Myc-$Usp25^{C178S}$ using PEI. After 24 hours post-transfection, cells were treated with 25 μM MG132 for six hours. The cells were lysed in RIPA lysis buffer (150 mM NaCl, 1% (w/v) TritonX-100, 1 mM EDTA, 0.5% (w/v) sodium deoxycholate, protease inhibitors cocktail (Roche), 50 mM Tris, pH 7.4) containing 100 mM N-ethylmaleimide (NEM) (Sigma-Aldrich). Ubiquitinated protein were precipitated using anti-Flag M2 affinity gel and analyzed by western blot.

Pulse-chase analysis

Pulse chase experiments were performed as previously described.[13,17,37] Briefly, either mock or virus-infected cells were grown in culture (50μM MG132 was added to the overnight culture medium where indicated), collected by trypsinization and starved for 30 minutes in methionine/ cysteine free DMEM at 37°C before pulse labelling. Cells were labelled for 10 minutes at 37°C with 10 mCi/ml [^{35}S]

methionine/cysteine (expressed protein mix; PerkinElmer) and chased for the indicated time points. At appropriate time points, aliquots were withdrawn and the reaction was stopped with 500 μl cold PBS. Cell pellets were lysed in Tris buffer containing 0.5% NP-40 and pre-cleared with agarose beads for one hour at 4°C. Immunoprecipitations were performed for three hours at 4°C with gentle agitation. Samples were eluted by boiling in reducing sample buffer, subjected to SDS-PAGE and visualized by autoradiography.

Western blot analysis

Cells were lysed in RIPA lysis buffer and the cell pellets were removed by centrifugation at speed of 14,000 rpm for 30 minutes at 4°C. Protein samples were prepared by mixing with 4 × Laemmli Sample Buffer (Bio-Rad) supplemented with β-mercaptoethanol (Sigma-Aldrich) and boiled at 95°C for 10 minutes. After SDS-PAGE, the proteins were transferred from the gel to PVDF membranes (Bio-Rad). Membranes were blocked with 5% skim milk in PBS supplemented with 0.1% Tween-20 (PBST) for one hour at room temperature and incubated overnight with primary antibodies diluted in 5% skim milk in PBST at 4°C. Membranes were washed three times, 10 minutes each with PBST, incubated with secondary antibodies for one hour at room temperature. Afterwards, membranes were washed three times, 10 minutes each with PBST. Positive immunostaining bands on the membranes were visualised using ECL Select Western Blotting Detection Reagent (GE Healthcare) and scanned by ImageQuant (GE Healthcare).

Fluorescence-activated cell sorting analysis

Cells were infected by viruses at a MOI of 5. Cell pellets were collected, fixed and permeabilized using Fix perm kit (BD PharMingen) according to manufacturer's instruction. Cells were washed three times with perm wash (BD PharMingen), followed by primary antibodies incubation for one hour and then secondary antibodies for 30 minutes on ice. The stained cells were analyzed by Attune NxT flow cytometer and the data analysis was performed with FlowJo.

Immunofluorescence assay

One day before the experiment, cells were seeded on coverslips pre-coated with Poly-L-Lysine (Sigma-Aldrich) at a density of 0.3 x 10^6 cells/well in 12-well plates. For viral entry assay, viruses were labelled with Octadecyl Rhodamine B Chloride (R18) (Thermo Fisher Scientific) according to manufacturer's instruction. Cells were infected by viruses at a MOI of 10. Coverslips with cells were collected and washed twice with PBS, followed by fixation with 4% formaldehyde for 30 minutes at room temperature. The cells were permeabilised with 0.5% Triton, followed by two washes with PBS. The cells were then blocked with 5% NGS for 30 minutes at room temperature. Primary antibodies at their appropriate dilution in PBS was added and incubated for one hour at room temperature followed by two washes with PBS. Secondary antibodies diluted in PBS was dispensed and incubated for 30 minutes at room temperature. After three washes, the coverslips were mounted on glass slides with DAPI Fluoromount-G (Southern Biotech) and kept at 4°C until visualization with confocal microscopy.

Immunoprecipitation and mass spectrometry

Both mock- and IAV-infected Flag-Usp25^{WT} cells were harvested and lysed with RIPA buffer after 24 hours post infection. Large-scale immunoprecipitations were performed on anti-Flag M2 affinity gel. Protein samples were resolved using SDS-PAGE with all protein stacked forming a band at the boundary between the stacking and resolving gel. The bands were visualized with Coomassie stain and gel lanes were separately cut into slices. Samples were trypsinized and subjected to Bruker timsTOF pro mass spectrometer for identification of candidates. MS/MS spectra were analyzed using Sequest algorithm searching a composite target-decoy protein sequence database. The target sequences comprised the Human UniProt FASTA database (April 2020) and protein sequences corresponding to the influenza H1N1 pandemic strain. Allowed criteria for searches required trypsin cleavage (two missed cleavage allowed), peptide mass tolerance of 20 p.p.m., variable oxidation of methionine residues and acetylation (Protein N-term), and static carbamidomethyl modification of cysteine residues. Confident proteins were identified using a target-decoy approach with a reversed database, strict false-discovery rate 1% at peptide and peptide spectrum matches level; minimum ≥1 unique peptide, ≥2 peptide spectral matches. Identified hits were further categorized into different biological pathways using Ingenuity Pathway Analyzes software.

Quantitation of cholesterol levels

Lipids were extracted with hexane/isopropanol (vol/vol, 3:2) from cells. The organic solvent containing the extracted lipids was evaporated under a gentle stream of nitrogen and used for measurement of total cholesterol (Amplex Red Cholesterol Assay Kit; Life Technologies).

QUANTIFICATION AND STATISTICAL ANALYSIS

For RT-qPCR, fold changes in cRNA, vRNA and mRNA expression were determined using ΔΔCt method relative to the values in control samples as indicated in figure legends, after normalisation to housekeeping genes. Results generated for protein expression, autophagosome and autolysosome/amphisome population, human anti-virus and proinflammatory chemokines responses were analyzed by ImageJ, FlowJo and Legendplex v8.0, respectively. Student's unpaired t test was performed to compare between two populations of data (e.g., control and sample) whereas two-way ANOVA was applied for multi sample comparisons. All data generated were from independent biological replicates where $n \geq 3$, each measured in technical duplicate or triplicates. Results have been presented as means ± standard deviation (SD). Statistical analyzes were performed in GraphPad PRISM 8.0. Pathway enrichment of hits from Usp25 interactome was performed using Ingenuity Pathway Analysis software.

Developmental Cell, Volume *58*

Supplemental information

Usp25-Erlin1/2 activity limits cholesterol flux

to restrict virus infection

Qi Wen Teo, Ho Him Wong, Tiaan Heunis, Viktoriya Stancheva, Asmaa Hachim, Huibin Lv, Lewis Siu, Julian Ho, Yun Lan, Chris Ka Pun Mok, Rachel Ulferts, and Sumana Sanyal

Supplementary Text

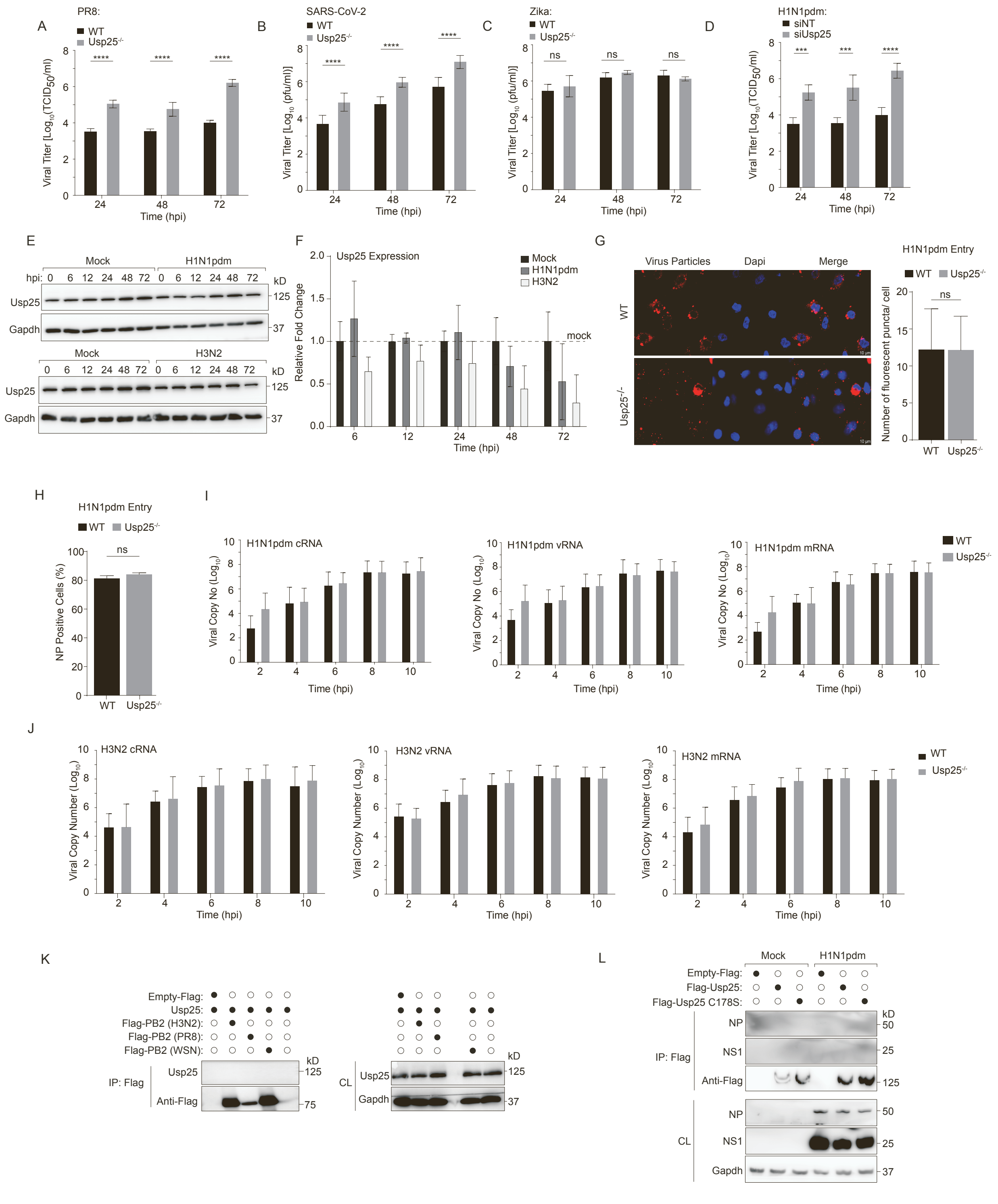

Figure S1. Usp25-deficiency does not affect viral entry and replication, related to Figure 1

(A-C) Viral titers measured from WT and Usp25$^{-/-}$ A549 cells infected with IAV PR8 (A), SARS-CoV-2 (B) and Zika (C) at the indicated timepoints (MOI = 0.1). Viral titers were determined by

plaque assay on MDCK and Vero cells respectively. Data are shown as means ± SD of three independent experiments. [****p<0.0001 analyzed by one-way ANOVA].

(D) Primary human bronchial epithelial cells from ATCC were treated with control siRNA (siNT) or Usp25 (siUSP25) and challenged with H1N1pdm (MOI 0.01). At indicated time points, viral titres were measured using TCID50/ml. Data are shown as mean ± SD of three independent experiments. [****p<0.0001 by one-way ANOVA].

(E) Western blot analysis of Usp25 protein expression upon IAV infection in WT A549 cells at the indicated timepoints; MOI = 0.01 used for multiple infection cycles; and MOI = 5 for single infection cycle. Gapdh was used as loading control. **(F)** Protein expression was quantified by densitometry and normalized to Gapdh levels. Data are shown as means of n=3 ± standard deviations (SD). Data analyzed by two-way ANOVA.

(G; ***left panel*****)** Immunofluorescence images of A549 cells infected with R18-labelled H1N1pdm virus (MOI=10). Viral particles are depicted in red. (*Right panel*) Viral entry was quantified by calculating the number of internalized viral particle per cell. Data are shown as means of n=50 ± standard deviation (SD). Data analyzed by Student's unpaired T test; ns: not significant.

(H) WT and Usp25$^{-/-}$ A549 cells were infected with H1N1pdm virus and the infected positive cells for NP were quantified by flow cytometry (MOI= 5). Data are shown as means of n=3 ± standard deviations (SD). Data analyzed by Student's unpaired T test; ns: not significant.

(I, J) cRNA, vRNA, mRNA levels of the Influenza M1 gene after H1N1pdm infection (I) or H3N2 infection (J) in A549 cells (MOI=5). Samples were collected at the indicated timepoints and were analyzed by real-time RT-qPCR. The specificities of the amplified products were confirmed by the melting curve analysis. Data are shown as means of n=3 ± standard deviation (SD). Data analyzed by two-way ANOVA.

(K) HEK-293T cell extracts, transiently co-transfected with Usp25 together with the indicated plasmids encoding the Flag-PB2 from H3N2, PR8, or WSN IAV strains were immunoprecipitated on anti-Flag M2 affinity beads. The immunoprecipitates (IP) were analyzed by immunoblotting (IB) with anti-Usp25 and anti-Flag antibodies. Transfected USP25 was detected by immunoblotting the cell lysates (CL) with anti-Usp25. Gapdh was used as loading control.

(L) H1N1pdm infected HEK-293T cell extracts, transiently transfected with Flag-Usp25 or Flag-USP25 C178S expression constructs were immunoprecipitated on anti-Flag M2 affinity beads. The immunoprecipitates (IP) were analyzed by immunoblotting (IB) with anti-NP and anti-NS1 antibodies. NP and NS1 expression were detected by immunoblotting the cell lysates (CL) with respective antibodies. Gapdh was used as loading control.

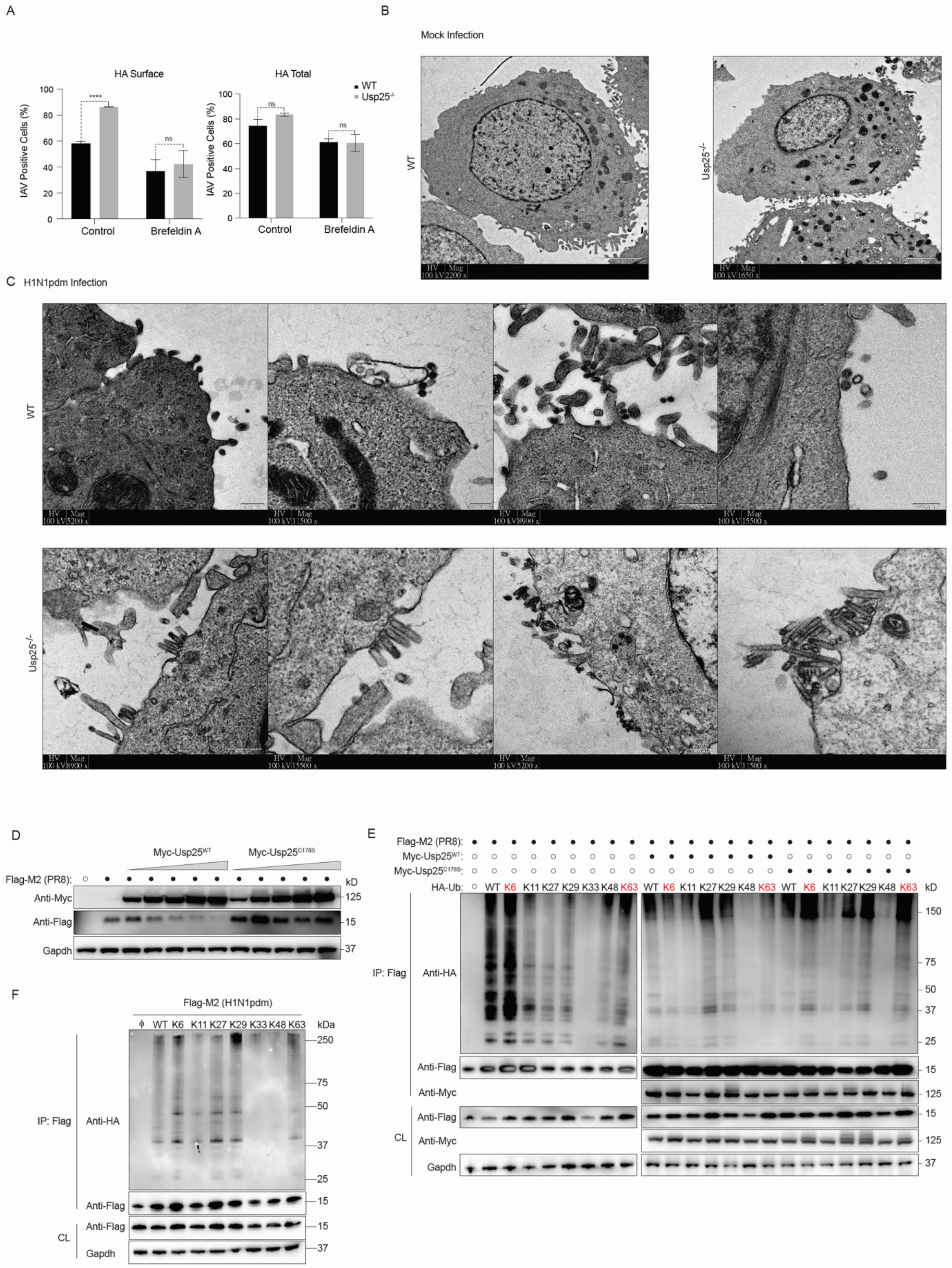

Figure S2. Usp25 alters M2-dependent budding of viral progenies, related to Figure 2

(A) IAV-infected WT and Usp25$^{-/-}$ A549 cells (MOI=5) were treated with Brefeldin A and total versus surface levels of viral HA was measure using flow cytometry. Data are shown as means of n=3 ± standard deviations (SD). Data analyzed by Student's unpaired T test was [**p<0.01, ***p<0.001].

(B, C) Negative stain EM of mock and H1N1pdm infected cells. (**D**) HEK293T cells co-transfected with Flag-M2 and Myc-Usp25^{WT} or Myc-Usp25^{C178S}. Expression of M2 and Usp25 were measured by immunoblotting. Anti-Myc was used to verified Usp25, and anti-Flag for M2. Gapdh was used as loading control**. (E)** HEK293T cells were co-transfected with Flag M2 and either myc-Usp25^{WT} or myc-Usp25^{C178S} along with HA-tagged ubiquitin (wild-type versus linkage specific constructs as indicated). M2 was immunoprecipitated on anti-Flag M2 affinity beads antibodies followed by immunoblotting with anti-HA to determine ubiquitylation. Anti-Myc was used to verified Usp25, and anti-Flag for M2. Gapdh was used as loading control. **(F)** Same as (E) using M2 from H1N1pdm IAV-strain.

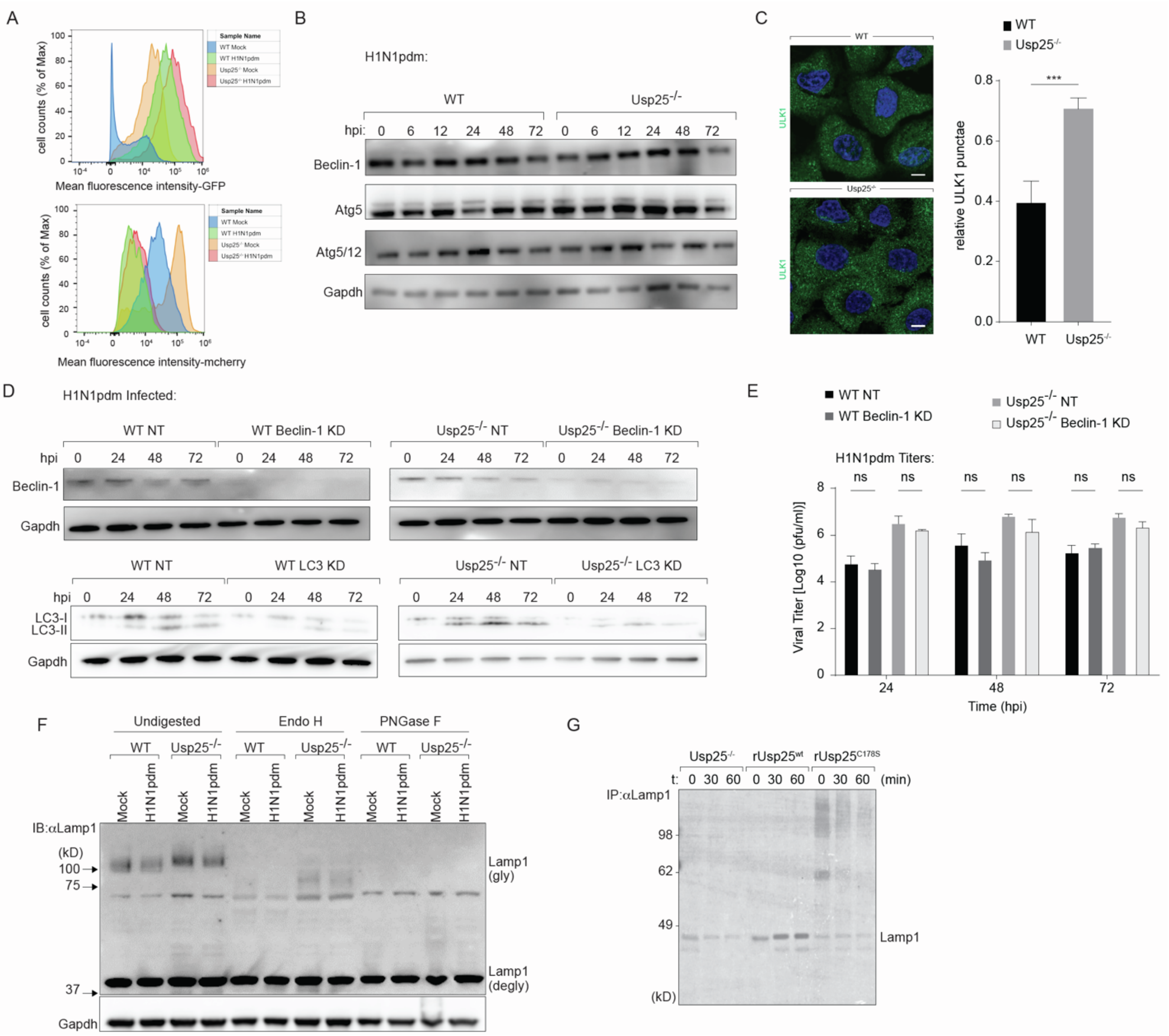

Figure S3. Usp25-deficient cells facilitate virus production via LC3 and Rab11 compartments, related to Figure 3

(A) Quantitation of mcherry and GFP populations of LC3 in mock and infected A549 cells. (**B**) WT A549 cells and Usp25$^{-/-}$ cells were infected with H1N1pdm (MOI=0.01). Total cell lysates were collected at the indicated time points, and protein expression of the autophagy markers were determined by Western blots. Gapdh levels measured as a loading control.

(**C**) ULK1 was visualized by confocal imaging in WT and Usp25$^{-/-}$ A549 cells. Quantitation of ULK1 punctae was calculated using "Analyze Particles" macro in Image J software. The quantitation was performed from three independent experiments with a sample size of 100 cells for each condition used to calculate statistical significance.

(**D**) Validation of Beclin1 and LC3 depletion was performed by immunoblotting in H1N1pdm-infected WT and Usp25$^{-/-}$ A549 cells at indicated timepoints. (MOI=0.01). Gapdh levels measured as a loading control.

(**E**) Viral titers from WT and Usp25$^{-/-}$ A549 cells depleted in Beclin1 at indicated timepoints were measured by plaque assay. (MOI=0.01). Data are shown as means of n=3 ± standard deviations (SD). Two-way ANOVA was used to analyze data [ns: not significant].

(**F, G**) Western blot analysis of Lamp1 glycosylation in WT and Usp25$^{-/-}$ A549 cells. Cells were infected with H1N1pdm (MOI= 0.01). Cell lysates were harvested and digested with either EndoH to cleave high-mannose and hybrid-mannose from *N*-linked glycoproteins or PNGase to remove all *N*-glycans for an hour. Gapdh was used as a loading control. (F) Usp25$^{-/-}$, Usp25^{WT} and Usp25^{C178S} were pulsed with [^{35}S]cysteine/methionine and chased for indicated time intervals. Lamp1 was immunoprecipitated on anti-Lamp1 antibodies, resolved by gel electrophoresis and detected by autoradiography.

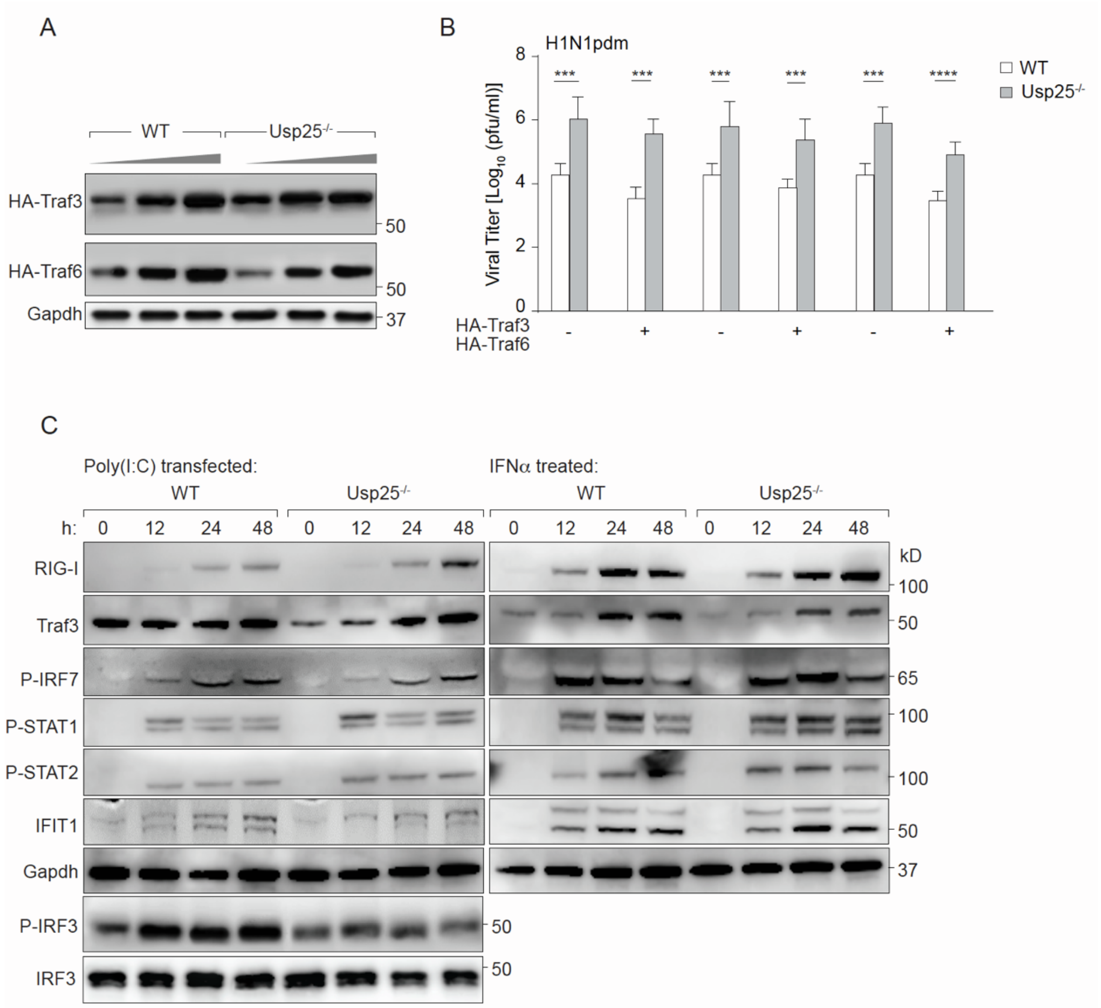

Figure S4. Usp25-deficient cells display attenuated immune responses, related to Figure 4

(**A**) Western blot analyses of Traf3 and Traf6 dose-dependent expression in the WT and Usp25$^{-/-}$ cells. Gapdh was used as loading control. (**B**) Viral titers were measured by plaque assay in cells from (A) infected with H1N1pdm (MOI=0.01) at 24 hours. **(C)** Western blot analysis of RIG-I and downstream effectors of the RIG-I pathway protein expression in WT and Usp25$^{-/-}$ A549 cells treated with either poly I:C or IFN-α at indicated timepoints. Gapdh was measured as loading control.

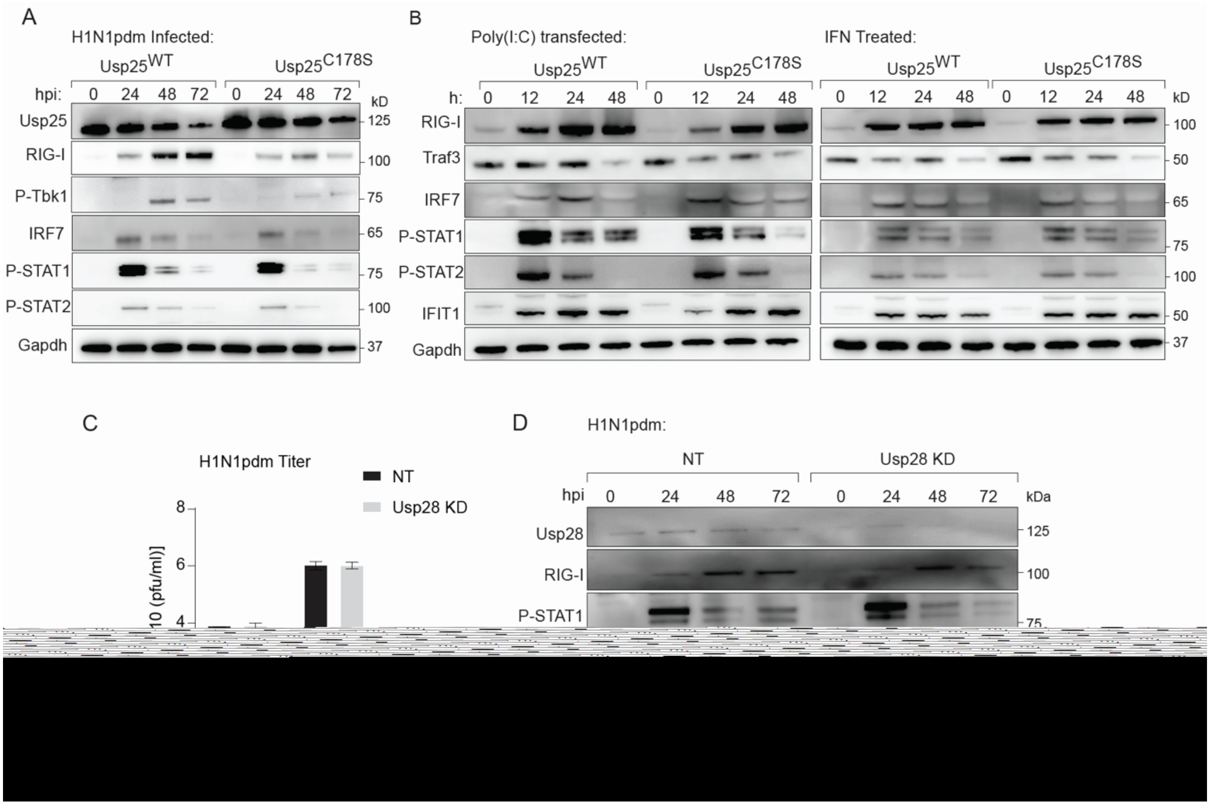

Figure S5. Catalytic activity of Usp25 is necessary for its antiviral function, related to Figure 5

(**A, B**) Western blot analysis of Usp25, RIG-I, and downstream effectors of the RIG-I pathway protein expression in H1N1pdm-infected (A) or either poly I:C or IFN-I treated (B) Usp25^{WT} and Usp25^{C178S} A549 cells. The cells were infected with H1N1pdm at MOI of 0.01. Gapdh was used as loading control.

(**C**) Viral titer of Usp28 knockdown (Usp28 KD) and non-targeting (NT) control A549 cells after H1N1pdm infection at the indicated timepoints (MOI=0.01). Viral titres were determined by plaque assay using MDCK cells. Data are shown as means of n=3 ± standard deviations (SD). Two-way ANOVA was used to analyze data.

(**D**) Western blot analysis of Usp28, RIG-I, phosphorylated STAT1, phosphorylated STAT2 and LC3 protein expression in H1N1pdm-infected NT and Usp28 KD A549 cells. The cells were infected with H1N1pdm (MOI=0.01). Gapdh was used as loading control.

captured on Neutravidin beads followed by re-IP on anti-influenza HA antibodies and detected by autoradiography.

(**F, G**) Western blot analyzes of Rab11, LC3I/II, Lamp2 and Cathepsin B expression in control (shNT) and shErlin1/2 A549 cells at indicated timepoints post H1N1pdm infection (MOI 0.01). Erlin1/2 depleted cells from (F) were treated with DMSO alone or Simvastatin (10μM) prior to H1N1pdm infection (MOI 0.01). Western blotting of proteins was performed in cell lysates prepared at indicated timepoints. Actin levels were measured as a loading control.

(**H**) Control and shErlin1/2 mCherry-GFP-LC3 A549 cells were infected with H1N1pdm (MOI 5). Quantification of autophagosome/autolysosome populations was performed by flow cytometry. Data are shown as mean± SD from 3 independent experiments. [*p<0.1, **p<0.01, ***p<0.001, ****p<0.0001, analyzed by one-way ANOVA].
